# Mechanistic Insights into the Charge Transfer Dynamics of Photocatalytic Water Oxidation at the Lipid Bilayer–Water Interface

**DOI:** 10.1021/jacs.2c06842

**Published:** 2022-10-17

**Authors:** Hongwei Song, Agnese Amati, Andrea Pannwitz, Sylvestre Bonnet, Leif Hammarström

**Affiliations:** † Department of Chemistry−Angstrom Laboratory, Uppsala University, Box 523, 751 20 Uppsala, Sweden; ‡ Leiden Institute of Chemistry, Leiden University, Einsteinweg 55, 2333 CC Leiden, The Netherlands; § Institute of Inorganic Chemistry I, Ulm University, Albert-Einstein-Allee 11, 89081 Ulm, Germany

## Abstract

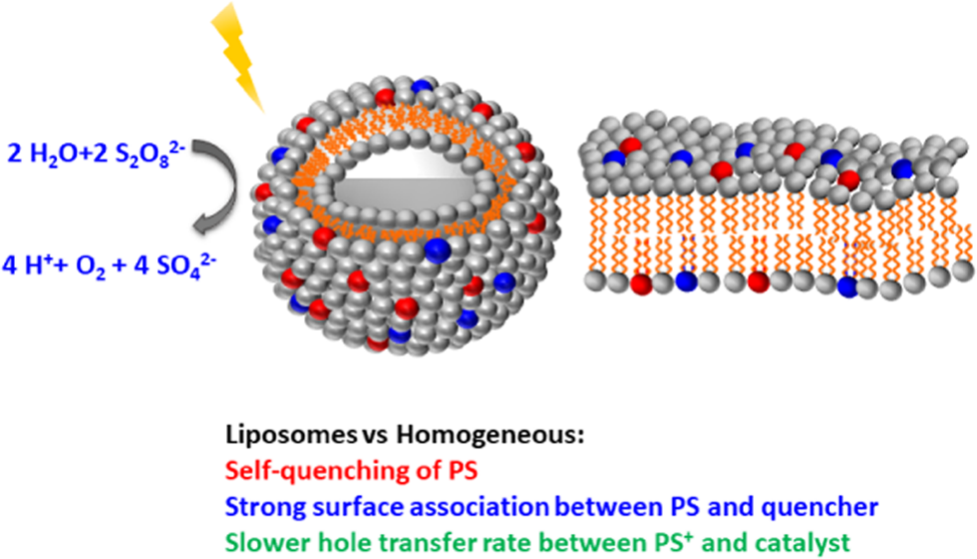

Photosystem II, the natural water-oxidizing system, is a large protein complex embedded in a phospholipid membrane. A much simpler system for photocatalytic water oxidation consists of liposomes functionalized with amphiphilic ruthenium(II)-tris-bipyridine photosensitizer (PS) and 6,6′-dicarboxylato-2,2′-bipyridine-ruthenium(II) catalysts (Cat) with a water-soluble sacrificial electron acceptor (Na_2_S_2_O_8_). However, the effect of embedding this photocatalytic system in liposome membranes on the mechanism of photocatalytic water oxidation was not well understood. Here, several phenomena have been identified by spectroscopic tools, which explain the drastically different kinetics of water photo-oxidizing liposomes, compared with analogous homogeneous systems. First, the oxidative quenching of photoexcited PS* by S_2_O_8_^2–^ at the liposome surface occurs solely via static quenching, while dynamic quenching is observed for the homogeneous system. Moreover, the charge separation efficiency after the quenching reaction is much smaller than unity, in contrast to the quantitative generation of PS^+^ in homogeneous solution. In parallel, the high local concentration of the membrane-bound PS induces self-quenching at 10:1–40:1 molar lipid–PS ratios. Finally, while the hole transfer from PS^+^ to catalyst is rather fast in homogeneous solution (*k*_obs_ > 1 × 10^4^ s^–1^ at [catalyst] > 50 μM), in liposomes at pH = 4, the reaction is rather slow (*k*_obs_ ≈ 17 s^–1^ for 5 μM catalyst in 100 μM DMPC lipid). Overall, the better understanding of these productive and unproductive pathways explains what limits the rate of photocatalytic water oxidation in liposomal vs homogeneous systems, which is required for future optimization of light-driven catalysis within self-assembled lipid interfaces.

## Introduction

Artificial photosynthesis aims at the direct generation of fuels from solar energy, which is a long-held goal for chemists and could contribute to covering the demand of globally increasing power consumption and the threatening situation of global warming related to burning fossil fuel.^1−3^ Photocatalytic water splitting into hydrogen (H_2_) and oxygen (O_2_) using sunlight is of significant importance for solar energy conversion. The overall process consists of an oxidative and a reductive half-reaction: water oxidation, which involves four-electron transfer step (2H_2_O → 4H^+^ + 4e^–^ + O_2_), is being considered particularly difficult compared with the two-electron reduction of protons (2H^+^ + 2e^–^ → H_2_).^4−7^ In nature, water oxidation takes place in photosystem II which is assembled within the thylakoid membranes in chloroplasts. Upon electronic excitation of the central chlorophylls (P680), subsequent excited state electron transfer to pheophytin, quinone (*Q*_A_) and then multielectron acceptor quinone (*Q*_B_), takes place, as well as hole transfer to a nearby tyrosine (Tyr_Z_), generating a tyrosine radical Tyr_Z_^**•**^, which then oxidizes the CaMn_4_ water oxidation catalyst.^8−11^ In biological photosynthesis, the thylakoid membrane plays a key role in the compartmentalization, spatial organization, and electronic coupling of the redox-active compounds, which altogether achieves efficient charge separation with low charge recombination rates, thereby resulting in accumulative charge separation of multiple redox equivalents on the CaMn_4_ cluster (holes) and *Q*_B_ (electrons), respectively.^8,12^

**Scheme 1:**
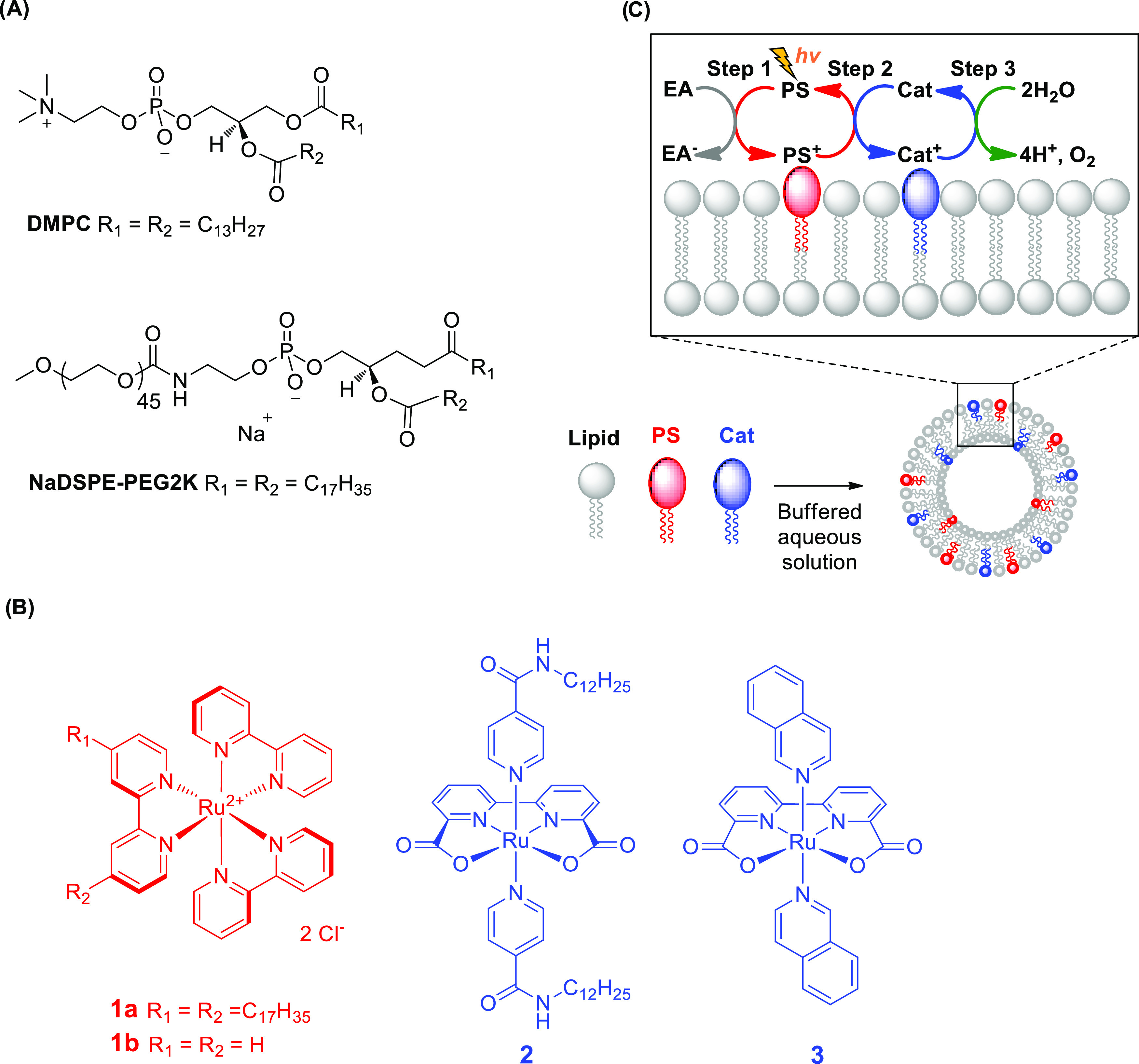
Chemical Structures of (A) Phospholipids (DMPC) 1,2-Dimyristoyl-*sn*-glycero-3-phosphocholine and (NaDSPE-PEG2K) 1,2-Distearoyl-*sn*-glycero-3-phosphoethanolamine-*N*-[methoxy(polyethyleneglycol)-2000] Sodium Salt, and (B) Lipophilic (**1a**, **2**) and Water-Soluble (**1b**, **3**) Water Oxidation Catalysts and Alkyl-Functionalized Ruthenium Tris-Bipyridine Potosensitizer Utilized in This Study. (C) Schematic of a Molecularly Functionalized Liposome System, with the Inset Highlighting a Simplified Representation of Different Electron Transfer (ET) Steps Occurring during Photocatalytic Water Oxidation on the Water–Membrane Interfaces Abbreviations: EA = electron acceptor, i.e., sodium persulfate (Na_2_S_2_O_8_), PS = photosensitizer, Cat = catalyst.

Important efforts have been dedicated to achieving artificial photosynthesis by mimicking natural photosynthesis using supramolecular systems, such as micelles,^13,14^ liposomes,^15−20^ polymers,^21−23^ and metal–organic frameworks.^6,24^ In liposomes, amphiphilic lipid molecules constituting the bilayer are oriented with their polar head groups toward the inner and outer aqueous solutions, while the hydrophobic chains form a nonpolar region between the two interfaces (see Scheme 1). In such systems, the bulk solution, the interface, and the membrane core are characterized by distinct dielectric constants, which offers opportunities to prearrange redox-active sites and modify chemical reaction rates and mechanisms, compared to homogeneous conditions.^20,25−27^ After pioneering but rare reports in the 1980s,^28^ several reports about photocatalytic water oxidation in liposomes have recently appeared. Typically, these photocatalytic systems consist of four components: the phospholipid that is the main component of the liposome membrane, an amphiphilic photosensitizer (PS), an amphiphilic catalyst, and a water-soluble sacrificial electron acceptor (SEA).^16,20,29−31^ Although such self-assembled systems have been utilized by several teams now, aiming essentially at maximizing product formation, little fundamental knowledge has been gathered on the effect of membrane-embedding on the rates of elementary electron transfer processes, where short-lived reactive intermediates are invoked.^32^

In this work, time-resolved transient absorption spectroscopy and photoluminescence quenching experiments were performed on a photocatalytic water oxidation liposomes system, to unravel detailed photophysical and kinetic information on hole transfer process during photocatalysis. The molecules used for the liposomal self-assembly are shown in Scheme 1. The amphiphilic ruthenium-based photosensitizer (**1a**) and catalyst (**2**) were embedded in liposome membranes composed of 1,2-dimyristoyl-*sn*-glycero-3-phosphocholine (DMPC), with 1 mol % 1,2-distearoyl-*sn*-glycero-3-phosphoethanolamine-*N*-[methoxy(polyethyleneglycol)-2000] sodium salt (NaDSPE-PEG2K) as sterically stabilizing agent, while Na_2_S_2_O_8_ acted as water-soluble sacrificial electron acceptor. For the comparison of the photocatalytic mechanism and rates in the two-dimensional assembly with that in a standard, homogeneous bulk aqueous solution, the water-soluble photosensitizer **1b** and the catalyst **3** were used. The mechanistic insights obtained in this study show how elucidating the elementary steps can provide valuable clues for reaction optimizations, allow us to draw conclusions on the limiting steps of the overall photocatalytic water oxidation, and to explain why these are different in the homogeneous and liposomal systems. In liposomes, the self-quenching of PS suggests that the direct absorpted light cannot fully be used to the following electron transfer and photocatalytic reactions. Stern–Volmer quenching studies show a corresponding decreased efficiency and also dynamic to static quenching from homogeneous to liposomes environment. In pH 4, the hole transfer rates between PS and Cat in liposomes become much slower than that in homogeneous environment. This work increases our understanding of liposome-based photocatalytic systems, which will benefit further work dedicated to the optimization of photochemical water splitting in interfacial, micro-heterogeneous systems.

## Results and Discussion

### Spectroscopic and Thermodynamic Properties of the Photosensitizers and Catalysts

**Figure 1:**
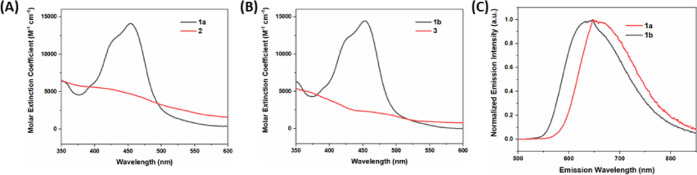
UV–vis spectra of (A) **1a** and **2** in liposomes (pH = 4) and of (B) **1b** and **3** in homogeneous solution. (C) Steady-state photoluminescence spectra of **1a** in liposomes (pH = 4) and **1b** in homogeneous phosphate buffer with 10% acetonitrile. Excitation wavelengths were fixed at 460 nm for the photoluminescence measurements.

Figure 1 shows the absorption and photoluminescence spectra of the liposome-embedded (panel A) and homogeneous components (panel B). The hydrophilic PS **1b** shows a visible absorption band at around 450 nm, and photoluminescence maximizing at around 640 nm (panel C). The amphiphilic PS **1a** in liposomal environment shows an ∼10 nm redshift of both absorption (460 nm) and photoluminescence (650 nm). The catalysts **2** and **3** show absorption bands that overlap with that of the PS, but with 3–5 times smaller extinction coefficients at the band maximum,^33^ and no detectable photoluminescence. Although some of the visible photons will be absorbed by the catalyst, their excited states are short-lived (∼20 ps)^33^ and are not expected to give significant photoproducts under the present conditions. The Ru^III/II^ potential for **1b** in water is *E*^0^ = +1.26 V vs NHE, while the Ru^III/II^ and Ru^IV/III^ potentials of **3** at pH = 0 are *E*^0^ +0.63 and +1.15 V vs NHE, repectively. The former apparent potential is pH-independent at pH < 5.5 but shows a Nernstian decrease of 59 mV/pH unit at pH > 5.5, indicating deprotonation of a coordinated water molecule, and the latter potential shows a 59 mV/pH unit decrease in the range pH = <1–13.^4,34^ The corresponding potentials for the amphiphilic **1a** and **3**, respectively, are expected to be similar, although the less polar membrane environment may destabilize the trivalent PS^+^ more than the monovalent Cat^+^. Thus, we expect that hole transfer to the Ru^II^ (Cat) and Ru^III^ (Cat^+^) catalyst are exergonic at pH = 0, with Δ*G*° = −0.63 and > −0.11 eV (the *E*°′ value at pH = 0), respectively, in the homogeneous system, and that the driving force in the liposomal system is similar or somewhat larger than that. If the hole transfer is coupled to deprotonation to water, the driving force for the initial step will be the same as the *E*°′ value shown in the Pourbaix diagram because the the water p*K*_a_ = 0 per definition, even if the subsequent proton dilution makes the overall process more favorable at higer pH values.

### Self-Quenching of Photosensitizer on Liposomes

**Figure 2:**
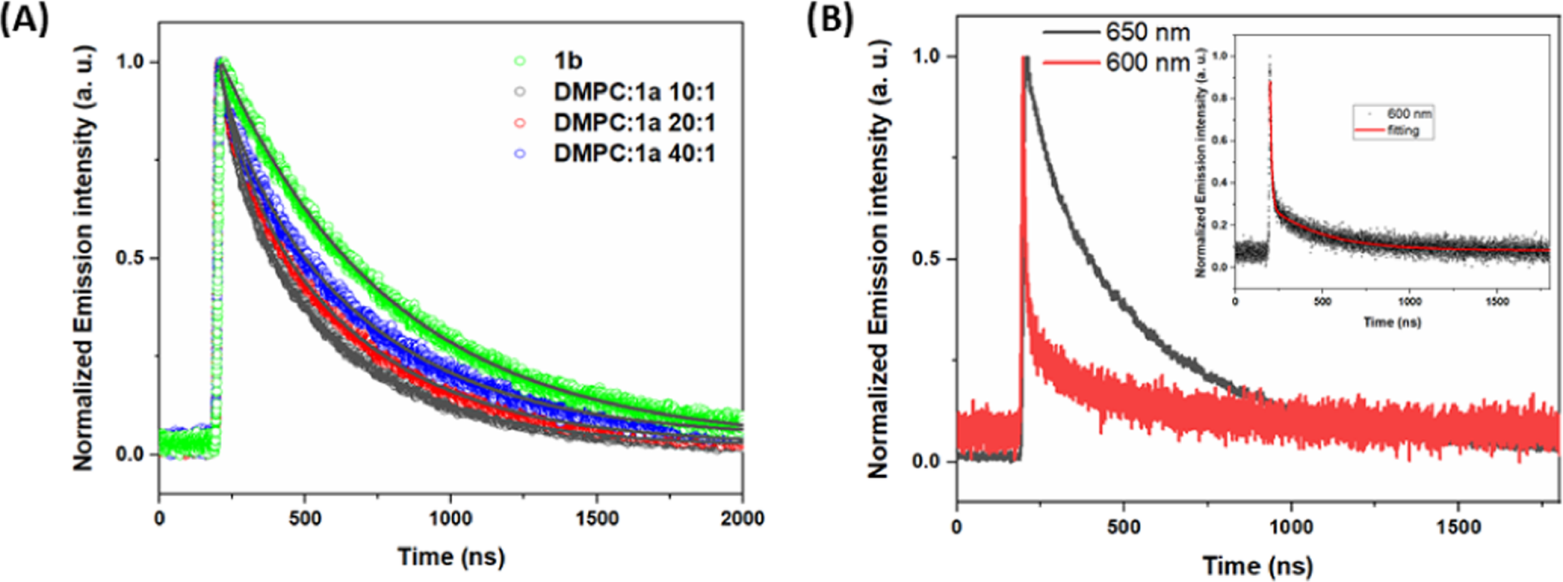
(A) Normalized photoluminescence lifetimes at 650 nm of **1a** in liposomes with different concentrations of **1a**, and of **1b** in homogeneous environment. (B) Normalized photoluminescence lifetimes of **1a** in liposomes at 600 and 650 nm; the inset is the fitting result of kinetic trace at 600 nm. Experimental conditions for liposomes: 10, 5, 2.5 μM **1a**, 100 μM DMPC, 1 μM NaDSPE-PEG2K in 50 mM phosphate buffer (pH = 7). Homogeneous conditions: 10 μM **1b** in 50 mM phosphate buffer (pH = 7). All solutions were purged with Ar before measurements at 20 °C. Excitation wavelength was fixed at 460 nm.

In the absence of the electron acceptor Na_2_S_2_O_8_, the single-wavelength time-resolved emission profile of 10 μM **1b** in water is single exponential, while for **1a** in liposomes it is not. Instead, a biexponential decay had to be used to fit the emission data in liposomes. The organization of PS **1a** at the surface of the bilayer influences both their local concentration and localization, which can in turn influence the photophysical emission properties. To investigate the local concentration effect, time-resolved emission studies with variation of local concentration of **1a** in the surface of DMPC were performed (see Figure 2). In this experiment the bulk lipid concentration remained constant (100 μM), while the total concentration of **1a** was varied (10, 5, and 2.5 μM). As shown in Figure 2A and Table 1, the relative amplitude of the short-lifetime component increased when the concentration of **1a** was increased (from 2.5 to 10 μM). Thus, the excited state lifetime decreases with the increasing surface concentration of PS in the liposomes. The observed biexponential decays can therefore be explained in terms of either self-quenching between excited and ground states, or triplet–triplet annihilation between two excited molecules of **1a**. To distinguish between these alternatives, measurements were repeated with variations of the energy of the excitation pulse, all at the same concentration of **1a** (10 μM). The emission decays showed laser-power-independent dynamics with excitation laser power in the range 20–50 mJ pulse^–1^ (Figure S1), which is not in agreement with a triplet–triplet annihilation process. Therefore, we can ascribe the short-lifetime component in the emission decays to self-quenching with ground-state neighbors of **1a** due to the high local concentration of the photosensitizer at the surface of DMPC liposomes, transiently forming dimers or larger aggregates. The anchoring of **1a** on liposomes provides a high local concentration of PS, but the resulting self-quenching of PS is not favorable for the whole photocatalytic water oxidation reaction because not all of the light absorbed by PS is used for the subsequent electron transfer reactions. We note that the lifetime of the short photoluminescence component (ca. 80 ns) is essentially constant with concentration. This can thus be attributed to the lifetime of the aggregated species (e.g., a weakly associated dimer). This is formed in an increasing fraction with increasing concentration, which explains the increasing relative amplitude of its photoluminescence.

**Table 1:** Lifetimes τ_1_ and τ_2_ Obtained from Fitting the Time-Dependent Phosphorescence Emission at 650 nm of **1a** in Liposomes at Various DMPC:**1a** Molar Ratios in 50 mM Phosphate Buffer (pH = 7)^a^

	τ_1_/ns (a_1_%)	τ_2_/ns (a_2_%)
**1b**	600 (100)	
molar ratio (DMPC:**1a**)		
10:1	78 (23.6)	412 (76.4)
20:1	81 (16.7)	458 (83.3)
40:1	80 (7.3)	496 (92.7)
10:1 (600 nm)	9 (95)	300 (5)

a The values found for **1b** in homogeneous solution in 50 mM phosphate buffer (pH = 7) and for **1a** at 600 nm at 10:1 molar ratio are also given.

We also note that the phosphorescence decay of **1a** in DMPC liposomes at 600 nm, in the blue edge of the band, is much faster than the one at the band maximum at 650 nm (Figure 2B). This also suggests that **1a** molecules in liposomes experience different environments and intermolecular interactions, possibly including dye aggregation, with somewhat different excited state energies, probably including energy transfer from higher to the lower energy sites. Recently, different dye locations in the liposome membrane was recently suggested in a similar system based on diffusion coefficient measurements.^35^

### Oxidative Quenching of the PS

The quenching of excited states of **1b** by electron transfer to S_2_O_8_^2–^ in homogeneous aqueous environments has been thoroughly studied in the literature.^36−38^ In brief, there are two possible pathways for the quenching reaction: either electron transfer within a ground-state ion-pair between **1b** and S_2_O_8_^2–^ (often denoted static quenching), or bimolecular electron transfer by diffusional encounter between an excited **1b** molecule and S_2_O_8_^2–^ (also called dynamic quenching).^39^ For dynamic quenching, the photoluminescence intensity (*I*) and lifetime (τ) are quenched to the same extent following eq 1
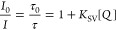
1where the zero subscripts in *I*_0_ and τ_0_ denote the intensity and lifetime in the absence of quenching, and the Stern–Volmer constant *K*_SV_ is equal to the product of the second-order quenching rate constant *k*_q_ and the unquenched lifetime τ_0_ (*K*_SV_ = *k*_q_ × τ_0_). For static quenching, the intensity is also quenched following eq 1, but with *K*_SV_ = *K*_A_, where *K*_A_ is the association constant between ground-state PS and quencher. In contrast, the lifetime of the unbound excited states is not affected (τ_0_ = τ).

**Figure 3:**
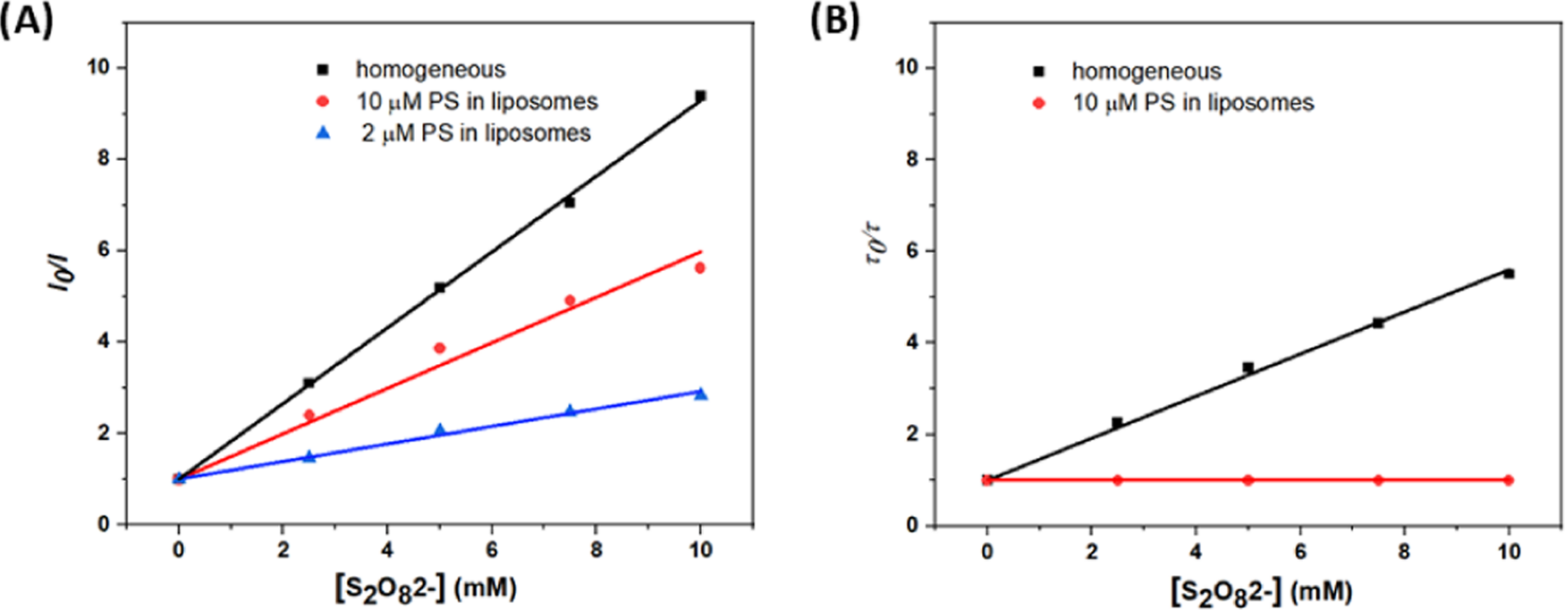
Stern–Volmer plots from photoluminescence quenching experiments, using (A) steady-state emission intensity and (B) lifetime data in homogeneous environment and liposomes. Experimental conditions in liposomes: 10 or 2 μM **1a**, 100 μM DMPC, 1 μM NaDSPE-PEG2K in 50 mM phosphate buffer (pH = 7). Homogeneous environment: 10 μM **1b** in 50 mM phosphate buffer (pH = 7); All solutions were purged with Ar before measurements at 20 °C. Excitation wavelength was fixed at 460 nm.

The quenching of the emission intensity and lifetimes of **1a** and **1b** with increased concentration of S_2_O_8_^2–^ are shown as a Stern–Volmer plot in Figure 3. The respective spectra and kinetic traces can be found in Figure S2. The different slopes of time-resolved emission (*K*_SV_ = 460 M^–1^) and steady-state data (*K*_SV_ = 830 M^–1^) in Figure 3 suggest that for the homogeneous case both dynamic and static quenching are simultaneously operative. There is no upward curvature in the *I*_0_/*I* plot as expected for a combination of both dynamic and static quenching,^40^ but in agreement with previous observations at high ionic strength (50 mM phosphate buffer in our case) the plots are linear.^36,38^ While there are some disagreements on interpretation in the literature, ion pairing is believed to be an important factor in the quenching mechanism. The details of the quenching mechanism in homogeneous solution is beyond the scope of the present study.

In liposomes, the emission intensity of the metal-to-ligand charge transfer (MLCT) excited state of **1a** was efficiently quenched by the electron acceptor S_2_O_8_^2–^; however, as shown in Figure S2E, the addition of S_2_O_8_^2–^ to a solution of DMPC/NaDSPE-PEG2K liposomes (100:1 μM) containing **1a** (10 μM) did not have an obvious effect on the emission lifetimes, i.e., the Stern–Volmer plot derived from time-resolved emission produced a flat line (Figure 3B). This is typical for static quenching^39^ and indicated the association of membrane-bound cationic PS and anionic acceptor S_2_O_8_^2–^ by electrostatic attraction, as is often observed in charged micelles or liposomes.^41−45^ The detailed nature of ion-pair species, with **1a**, DMPC, S_2_O_8_^2–^ and their counterions (Na^+^ and Cl^–^) and the buffer ions is likely to be a complex distribution of various associated species. In one limiting case, due to the equal and oppositely charged reactants, one S_2_O_8_^2–^ is associated to one PS molecule at the surface of liposomes, similar to ion-pair species in homogeneous solution. In the other limiting case, the negatively charged acceptor would be attracted by the collective electric field from several positively charged PS at liposomes surfaces.^27^ The slopes of Stern–Volmer plots (Figure 3A) indicated a change of apparent association constant from *K*_A_ = 200–500 M^–1^ when going from 2 to 10 μM **1a** in 100 μM DMPC. Thus, the higher the surface concentration of PS, the larger the fraction of bound persulfate, and the higher the probability that each excited PS is quenched. This result indicates a cooperative effect of the electric field from several cationic PS in associating with S_2_O_8_^2–^ quenchers. This also means that the Stern–Volmer slopes cannot be directly translated into a bimolecular association constant.

**Figure 4:**
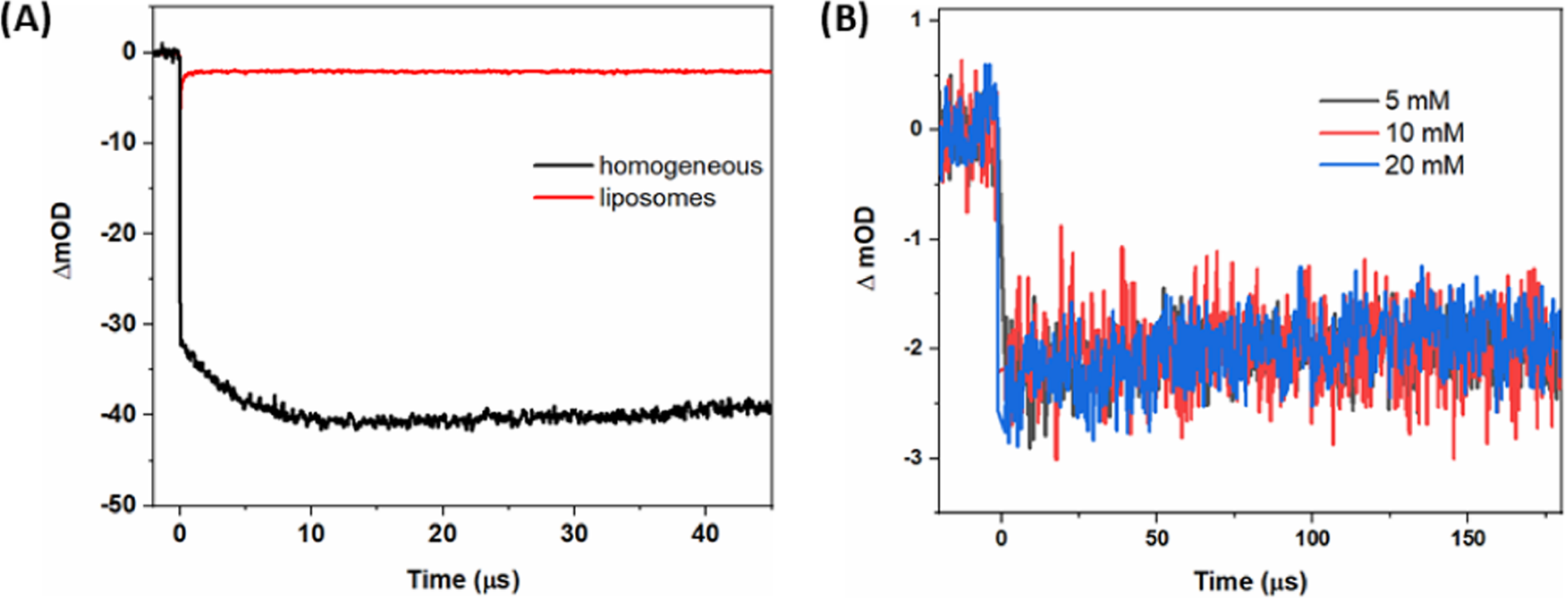
Transient absorption traces at 450 nm after laser flash excitation of (A) photosensitizer **1a** in liposomes and **1b** in homogeneous solution, with 5 mM Na_2_S_2_O_8_, and (B) photosensitizer **1a** in liposomes and 5, 10, or 20 mM Na_2_S_2_O_8_. Experimental conditions in liposomes: 10 μM **1a**, 100 μM DMPC, 1 μM NaDSPE-PEG2K in 50 mM phosphate buffer (pH = 7); Homogeneous environment: 10 μM **1b** in 50 mM phosphate buffer (pH = 7). All solutions were purged with Ar before measurements at 20 °C. Excitation wavelengths were fixed at 460 nm.

The photoluminescence experiments show that with appropriate concentrations, the excited states PS* can be efficiently quenched. We applied transient absorption spectroscopy to quantify the amount of catalytically relevant oxidized PS molecules (PS^+^) generated. In laser flash photolysis experiments, PS^+^ is rapidly generated on the time scale of 0.1–1 μs by photoreaction of PS* with the electron acceptor, and the formation of PS^+^ is detected as a bleach of the MLCT band, at 450 nm for **1a** and **1b** (Figure S3).^46,47^ In the experiment illustrated in Figure 4, aqueous solutions containing 5 mM Na_2_S_2_O_8_ and either liposomes at 10 μM **1a**, 100 μM DMPC, 1 μM NaDSPE-PEG2K, or homogeneous environment with 10 μM **1b**, in 50 mM phosphate buffer (pH = 7), were excited with an 8 ns laser pulse at 460 nm. In homogeneous environment, the increase of the PS^+^ signal clearly exhibited a prompt (<1 μs) and a slower component to the bleach amplitude (<10 μs, Figure 4A). According to the work of Scandola and co-workers,^48,49^ the fast component corresponds to the primary oxidative quenching photoreaction: Ru(bpy)_3_^2+*^ + S_2_O_8_^2–^ → Ru(bpy)_3_^3+^ + SO_4_^2–^ + SO_4_^**•**–^, and the latter to the secondary dark reaction: Ru(bpy)_3_^2+^ + SO_4_^**•**–^ → Ru(bpy)_3_^3+^ + SO_4_^2–^. Similar to their work, our data shows a somewhat smaller amplitude for the dark phase than the prompt reaction, for reasons that are not clear at present. After ∼10 μs, the bleach remained almost constant for at least hundreds of ms at pH = 7. For liposome samples, there were two obvious differences in the generation of oxidized PS in comparison to that in homogeneous solution: (i) the initial amplitude for the formation of PS^+^ was clearly 1 order of magnitude lower (2 mOD vs 32 mOD) and (ii) there was no secondary dark reaction by SO_4_^**•**–^ (0 mOD vs 10 mOD). By considering both direct photoquenching and secondary dark reaction, for liposomes system the overall quantum efficiency for PS^+^ generation was almost 20 times lower (2 mOD vs 42 mOD) than that in homogeneous conditions. This means that only a small portion of oxidative quenching processes in the liposomes results in the formation of long-lived PS^+^. This could in principle be due to an unidentified quenching mechanism induced by S_2_O_8_^2–^ which is operating in parallel to electron transfer, but we fail to see what mechanism that could be. Instead, the results suggest extensive charge recombination in the solvent cage, i.e.*,* that the initial products Ru^3+^ and S_2_O_8_^•3–^ recombine before they separate, and before S_2_O_8_^•3–^ dissociates into SO_4_^2–^ + SO_4_^•–^. We are not aware of previous reports of charge recombination between persulphate and a quenched triplet photosensitizer. Rapid recombination in the solvent cage before separation has been suggested to occur from the tertracationic Zn(II)-porphyrin singlet state quenching, while cage escape from the triplet state quenching was quantitative.^50^ In the present case, the initial electron transfer products are oppositely charged, and it is conceivable that product separation is hindered by the electrostatic attraction at the surface of liposomes, thus resulting in fast charge recombination.

In the photocatalytic water oxidation reaction, the quantum yield of oxidative quenching of PS by S_2_O_8_^2–^ is obviously important for the next steps to occur, as it is the primary step of the photocatalytic mechanism. In liposomes, this quenching reaction seems to be less efficient and becomes the bottleneck of the whole photocatalytic mechanism. A logical way to increase the oxidative quenching efficiency of PS* excited state would be to increase the concentration of quencher, Na_2_S_2_O_8_. However, as shown in Figure 4B, over the range of Na_2_S_2_O_8_ concentration tested (5, 10, and 20 mM) the amount of PS^+^ generated remained unchanged, indicating that the yield of long-lived oxidizing equivalents following quenching was independent from the bulk Na_2_S_2_O_8_ concentration. However, it should be noted that the emission quenching efficiency with 10 μM PS was ca. 77% already at 5 mM quencher (*I*_0_/*I* ∼4.3; Figure 3A). Only a small further increase in the yield of PS^+^ at higher concentrations of S_2_O_8_^2–^ may be expected (*I*_0_/*I* > 5.5 at 10 mM quencher, i.e., 82% quenching efficiency; Figure 3A), and this small increase may not be significant within the uncertainty of the experiment.

In summary, the data shows that the efficiency of PS* quenching is rather high, but the resulting free PS^+^ is produced with very poor quantum yield (∼5%). This number is in good agreement with the estimated initial quantum yield of ∼7% for a similar liposomal system when monitoring product photoaccumulation during steady-state irradiation.^16^

### Hole Transfer between Oxidized Photosensitizer and Water Oxidation Catalyst at pH 7 and pH 4

After the initial oxidative quenching step to form PS^+^ (step 1 in Scheme 1C), the next important reaction is hole scavenging, i.e., electron transfer from catalyst to PS^+^ (step 2 in Scheme 1C). Such a process is often a limiting step in the efficiency of dioxygen production because it is responsible for regenerating PS and allowing for the accumulation of holes on the catalyst.^5,49^ Flash photolysis measurements with the complete system in homogeneous environment and with liposomes were carried out to compare the kinetics of this step and assess its efficiency. Under neutral conditions (pH = 7, Figures S4D and S5B), the experiments quickly lead to the formation of a new absorption band at 690 nm, which is stable in the dark and that is characteristic for an oxo-bridged ruthenium trimer,^51^ or dimer^52^ formed from the Ru(III) state of this catalyst. Previous reports showed that the ruthenium dimer/trimer formation can be prevented using slightly acidic water,^51^ which led us to run new flash photolysis experiments at pH = 4 (Figures S4C and S5A).

**Figure 5:**
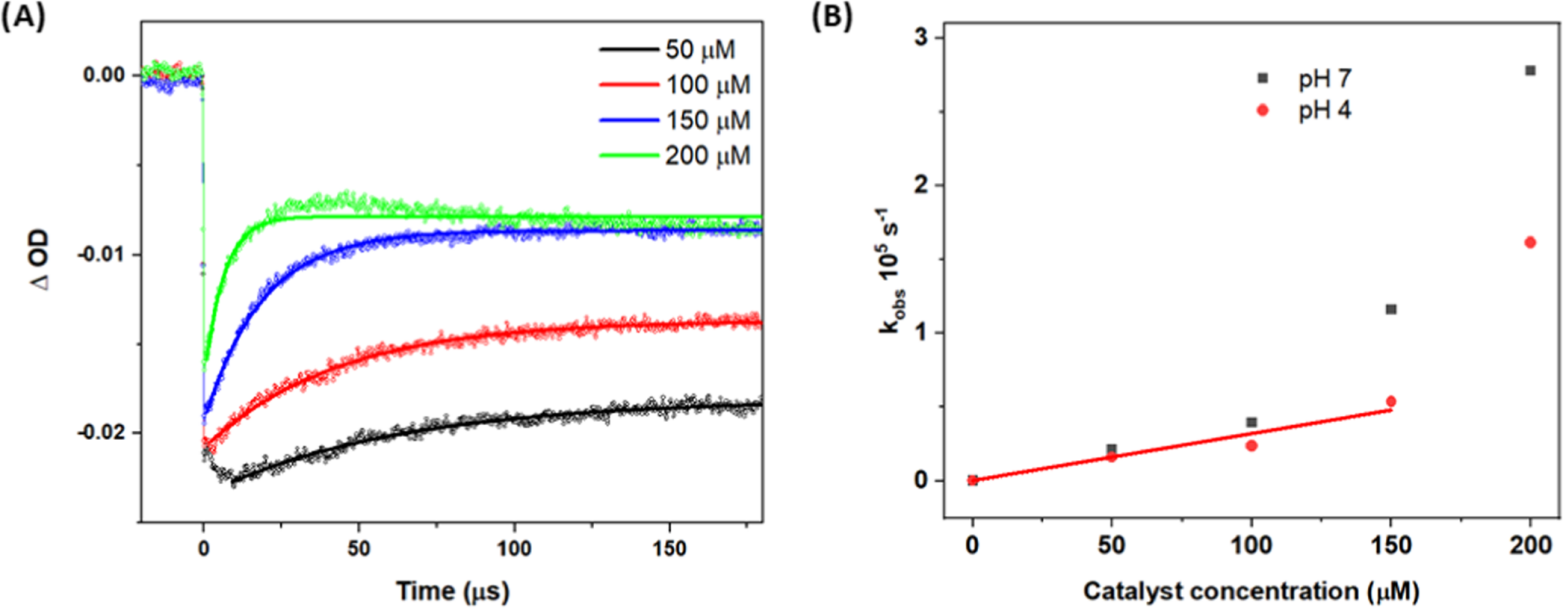
(A) Transient absorption traces at 450 nm after laser flash excitation of homogeneous, aqueous solutions containing 20 μM **1b**, 5 mM Na_2_S_2_O_8_, 50 mM phosphate buffer (pH = 4) and different concentrations of catalyst **3** (50, 100, 150, or 200 μM). The figure also includes monexponential fits yielding *k*_obs_. Excitation with 8 ns 460 nm pulsed light. (B) Observed pseudo-first-order rate constants (*k*_obs_) for electron transfer from **3** to PS vs concentration of **3**, at pH = 4 and 7. A linear fit to the data at pH = 4 with [**3**] = 0–150 μM gave a hole transfer rate constant *k* = 3.2 × 10^8^ M^–1^ s^–1^.

To follow the hole transfer process in homogeneous solution, we repeated the experiments of Figure 4A but at pH = 4 and in the presence of catalyst **3** (Figure 5); the initial photoreactions of PS and S_2_O_8_^2–^ do not change between pH = 4 and 7. The ground-state bleach of **1b** recovers with a rate that increases with increasing concentration of **3**. This observation can be attributed to the hole transfer process **1b**^**+**^ + **3** → **1b** + **3**^**+**^. As the concentration of **3** was increased, besides speeding up the disappearance of **1b**^**+**^, the oxidative formation of **1b**^**+**^ by SO_4_^**•**–^ also became less evident because of the simultaneous hole transfer to **3**, and because the SO_4_^**•**–^ radical started to oxidize **3** instead of **1b**. The bleach recovery at 450 nm was not complete even with the highest catalyst concentration employed (200 μM) because oxidation of the catalyst leads to a bleach of the catalyst absorption band that has a smaller extinction coefficient but a similar spectral range as that of the PS.^25^ The process was complete within ∼30 μs with 200 μM catalyst (*k*_obs_ = 1.7·10^5^ s^–1^ from a single-exponential fit; the concomitant dark oxidation by SO_4_^**•**–^ causes some deviation from a single exponential), but with 50–100 μM catalyst the reaction was much slower (*k*_obs_ ≤ 0.3 × 10^5^ s^–1^). This is a much stronger dependence than expected for a reaction first-order in [3]. We scrutinized the UV–vis absorption spectra of 20 μM **1b** with different concentrations of catalyst **3** (Figure S4E,F) and found that also at pH = 4 and [**3**] a clear dimer/trimer band is visible, while at [**3**] ≤100 μM, this is not seen. The presence of very reactive dimer/trimer at higher concentrations of **3**, already before irradiation and at pH = 4, can explain the strong increase of *k*_obs_ because of its lower potential for oxidation (see below). It is clear that the catalyst behavior is more complex than previously reported. We estimated the rate constant for hole transfer to monomeric catalyst **3** from the data at 0–150 μM to *k* = 3.2 × 10^8^ M^–1^ s^–1^.

In liposomes, the catalyst was exposed to air during liposome preparation (3–4 h), and we suspected that oxidation of **2** to the Ru^III^ state might occur. Therefore, we determined the oxidation state of the catalyst in liposomes before laser flash photolysis experiments by UV–vis absorption spectroscopy (Figure S6). To avoid the overlapping absorption of PS, liposomes were prepared with 100 μM DMPC and 5 μM amphiphilic catalyst **2** at pH = 4. By comparison with the published molar extinction coefficients^4,34^ almost 75% of amphiphilic catalyst **2** is in the Ru(III) state and 25% in the Ru(II) state before transient absorption spectral measurements. As the liposome preparation procedure is identical whether the PS is present or not, these results in the absence of PS should be representative also for the oxidation state of **2** in the photocatalytic liposomes containing **1a**. Similarly to the homogeneous solution, at pH = 4 in liposomes containing 10 μM **1a**, 5 μM **2**, 5 mM Na_2_S_2_O_8_, in 50 mM phosphate buffer, even though the catalyst was mainly found in the Ru(III) state, no formation of a catalyst dimer/trimer band at 690 nm no band was observed (Figure S5A). The dimer/trimer was however apparent in liposomes at pH = 7 (Figure S5B).

**Figure 6:**
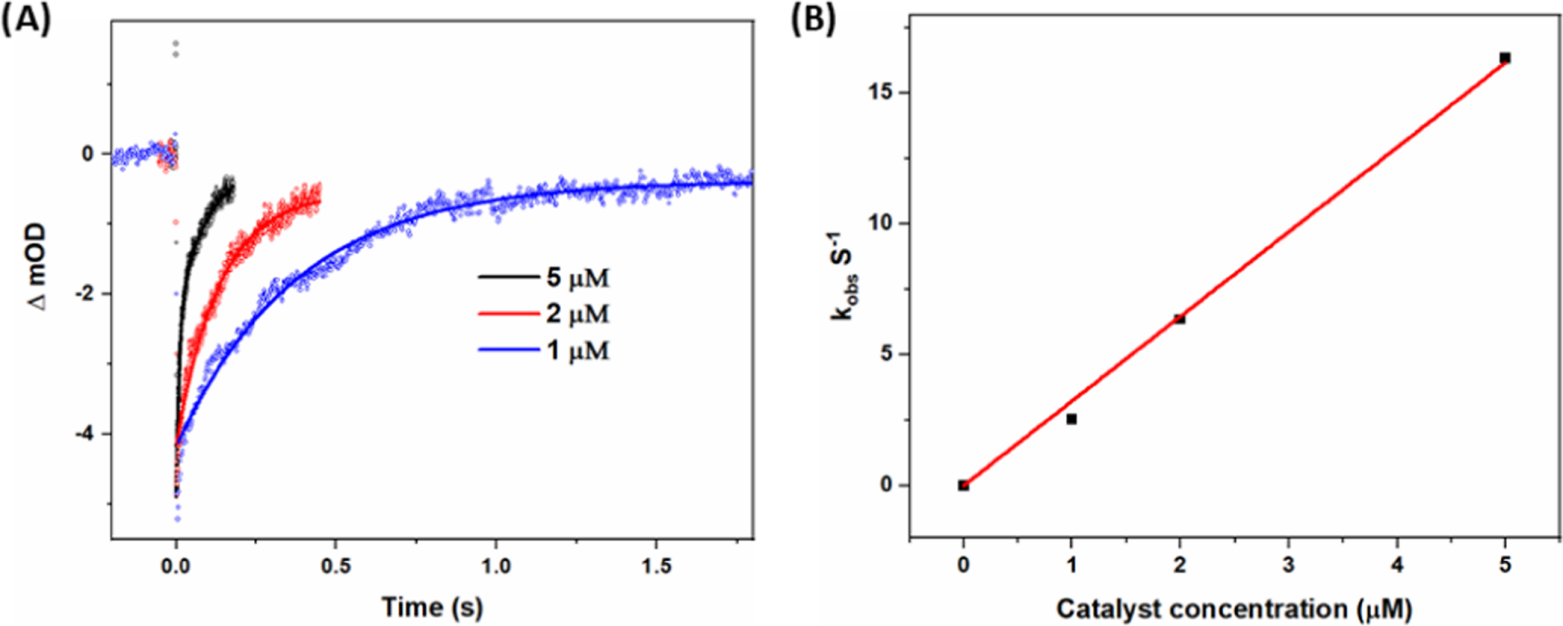
(A) Transient absorption traces at 450 nm after laser flash excitation of aqueous liposome solutions containing 10 μM **1a**, 1, 2, or 5 μM **2**; 5 mM Na_2_S_2_O_8_; and 10 μM DMPC with 1 μM NaDSPE-PEG2K in 50 mM phosphate buffer at pH = 4. Excitation with 8 ns 460 nm pulsed light. Black, red, and blue lines are biexponential fits, the fitting parameters are shown in Table S1. (B) Pseudo-first-order rate constants (weighted averages) vs concentration of **2** from the fits in panel (A). We subtract the rate without catalyst when calculating the hole transfer rates.

In the liposomes with both **1a** and **2** at pH = 4, electron transfer from **2** (1–5 μM) to PS^+^ occurred on the millisecond time scale, much faster than in the absence of catalyst (Figure S7A). The use of higher concentrations of **2** was not possible due to clogging of the extrusion membrane during liposome preparation. The traces were fitted with a biexponential function; this most probably reflects the micro-heterogeneous environment where reactants can be located in different parts of the lipid bilayer. In addition, with ca. 0.5 μM PS^+^ formed per flash, the catalyst concentrations cannot be assumed constant for 1 and 2 μM of **2**, but the biexponential character did not decrease with increasing concentration, so we think this effect is smaller than that of the intrinsic heterogeneity. Judging from the weighted average pseudo-first-order rate constant *k*_obs_, the reaction is first order with respect to the concentration of **2** (Figure 6A,B). This analysis gives a second-order rate constant for electron transfer from **2** to PS^+^ of ∼3 × 10^6^ M^–1^ s^–1^ based on bulk concentrations of **2**, which is extremely low. It is likely that it is rather the surface concentration ([**2**]/[DMPC] ratio) that is important for the rate, but light-scattering problems at higher [DMPC] and low signals at lower [PS] prevented a meaningful variation of the [**2**]/[DMPC] ratio to prove that point. Nevertheless, at 5 μM **2** in 100 μM DMPC and with a DMPC headgroup area of 60–65 Å^2^,^52^ there is on average one catalyst molecule per 12–13 nm^2^. Yet, the hole transfer reaction of PS^+^ to **2** is extraordinarily slow, with *k*_obs_ ≈ 17 s^–1^ at 5 μM **2**. Even if diffusion is slower in liposomal membranes than in water, a diffusion-controlled reaction would have shown a half-life below 1 μs at these surface concentrations.^27^ To figure out if the observed reaction of PS^+^ is mainly with the Ru(II) or Ru(III) form of the catalyst, control measurements were performed in homogeneous solutions, containing 20 μM **1a**, 100 μM **3**, and 5 mM Na_2_S_2_O_8_, that were exposed to air for different times, followed by purging with argon to remove oxygen, before the laser flash experiments started (Figure S8). As the air exposure time increased and the fraction of catalyst in the Ru(II) decreased, the reaction rate slowed down substantially. This showed that the Ru(III) state of the catalyst reacts more slowly with PS^+^ than the catalyst Ru(II) state. This difference can be expected from the much smaller driving force for oxidation of the Ru(III) state, compared to that of the Ru(II) state (Δ*G*° = −0.11 and −0.63 eV, respectively, see above). If a similar relation holds in liposomes, this results suggest that the 25% of catalyst **2** that is still in the Ru(II) state is dominating the liposomal photocatalytic reaction, and that the Ru(III) state reacts much slower than that. Thus, the experimentally estimated hole transfer rate constant 3 × 10^6^ M^–1^ s^–1^ would belong to the Ru(II)-to-Ru (III) oxidation of the catalyst **2**, and as the Ru(II) is only 25% of the total concentration of **2**, the value should rather be ∼1 × 10^7^ M^–1^ s^–1^.

In contrast, the same liposomal photocatalytic system at pH = 7 showed a dramatic increase in the observed pseudo-first-order rate constant when the catalyst concentration was increased from 1 to 5 μM from *k*_obs_ = 1.3 to *k*_obs_ = 4 × 10^3^ s^–1^, as shown in Figure S7. For the latter solution, there is 0.3 μM catalyst dimer/trimer present even before flash photolysis experiments started.^51^ At the liposome interface, the local catalyst concentration is higher than for **3** in homogeneous solution, and formation of dimer/trimer is facilitated. From the data in Figure S7, it seems that hole transfer to the dimer/trimer is very rapid, compared to the monomeric Ru^II^ species. Indeed, according to studies of a similar Ru(bda) complexs,^53^ monomer oxidation (Ru(II) to Ru (III)) is a single electron transfer process and the Pourbaix diagram shows that Ru(II) to Ru (III) is pH-independent from pH 1 to pH 7 with a potential *E* vs SCE (V) = 0.45. For the oxo-bridged dimer/trimer, the oxidation is a proton-coupled electron transfer process and at pH 7, the potential *E* vs SCE (V) = 0. Therefore, the dimer/trimer is more easily oxidized than the monomer.

### Photocatalytic Water Oxidation

The turnover frequency (TOF) is a measure of the reaction rate, and thus a key parameter for photocatalysis. It must be emphasized, however, that the TOF depends strongly on the type of experiment (chemical oxidant or photocatalytic conditions), the type of light (laser flash or continuous irradiation) as well as various experimental parameters, such as light intensity, buffer and concentration of the different reactants. Thus, sometimes comparisons of TOF between different conditions can be misleading, as different steps of the overall process can limit the observed TOF. In light-driven water oxidation reaction, the intrinsic water oxidation at the catalyst is often assumed to limit the TOF. In fact, three main processes are involved and each can limit the overall rate of dioxygen production: (1) the rate of step 1 in Scheme 1, i.e., the rate of PS^+^ generation, which is determined by the rate of photon absorption and the quantum efficiency of PS^+^ generation in the reaction of PS* with the electron acceptor; (2) the rate of step 2 in Scheme 1, i.e., of electron transfer between the catalyst and PS^+^; or (3) the rate of step 3 in Scheme 1, i.e., of catalytic water oxidation by the oxidized catalyst to yield molecular oxygen. The TOF is in fact equal to the rate of photon absorption multiplied by the quantum yield of product formation, and it is clear that the latter parameter is not a simple function of the rate of an individual reaction step.

Photocatalytic water oxidation in the present homogeneous and liposomal systems at pH = 7 was already studied extensively, with variation of the reaction conditions to identify the limiting step for the overall TOF.^16^ In homogeneous solution of **1b** and **3** with persulfate, it was found that electron transfer between catalyst and PS^+^ (step 2) was limiting the overall TOF. In liposomes with **1a** and **2**, however, membrane anchoring caused a large decrease in the quantum yield of oxidative quenching of PS by the electron acceptor (from 180% in homogeneous conditions to 7.3% in liposomes) and step 1 was concluded to be the rate-limiting step of photocatalytic water oxidation reaction.^16^ In the present work, the transient absorption measurements support the previous conclusion that the quantum efficiency of oxidative quenching of PS is ca. 20 times lower in liposomes than that in homogeneous environment. However, as has been discussed above, the hole transfer in liposomes at pH = 4 (*k*_obs_ ≈ 17 s^–1^ at [**2**] = 5 μM; 1st order rate dependence on [**2**]) is several orders of magnitude slower than that in homogeneous solution (*k*_obs_ ∼ 2 × 10^4^ s^–1^ at [**3**] = 50 μM; 1st order rate dependence on [**3**]). We tentatively explain the slower hole transfer rate in liposomes, in spite of a high (1:20) molar ratio between catalyst and DMPC, to the fact that the headgroup of the catalyst is more hydrophobic than that of **1b**, and may thus reside deeper in the liposome membrane. It may at first seem that in liposomes, except for the lower quantum efficiency of oxidative quenching of PS, the TOF may be limited by the slow hole transfer reaction between PS^+^ and the catalyst. Surprisingly, the oxidized photosensitizer, PS^+^, is kinetically very stable in slightly acidic water, however, and since all persulfate-generated radicals have already been consumed, the rate of charge recombination is exceptionally low. Therefore the yield of hole transfer to the catalyst can be very high, in spite of the slow rate. It should also be high enough to keep up with the inefficient generation of PS^+^; the PS is excited at most once per second under full sun irradiation, and the quantum yield of PS^+^ generation is at best 1/20 (5%, see above). Thus, PS is regenerated from PS^+^ much faster than PS is oxidized to PS^+^. This means that step 1 is still limiting the overall TOF. As a caveat, we should state that the full catalytic cycle of step 3 in Scheme 1 involves several catalyst oxidation steps for which we have no kinetic information, but as also the previous study of photocalytic experiments arrived at this conclusion, we believe it is correct.

**Figure 7:**
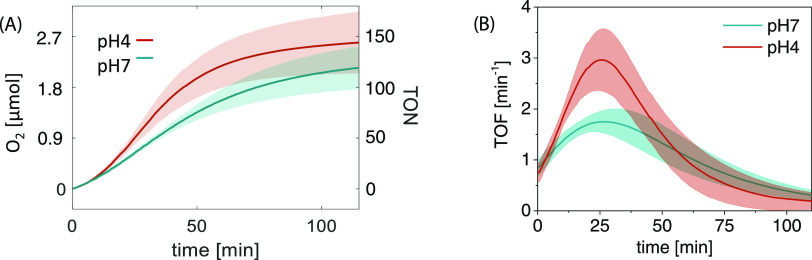
(A) TONs of photocatalytic oxygen evolution at different pHs vs irradiation time. Each curve is the mean of the data fitting of three replicate experiments (see the Supporting Information for raw data), and the shaded areas show the standard deviations for each series. Conditions: 100 μM DMPC, 1 μM NaDSPE-PEG2K, 5 μM **2**, 10 μM **1a**, 5 mM Na_2_S_2_O_8_ in 3.5 mL of phosphate buffer (50 mM, pH = 7.1 or = 4), λ_irr_ = 450 nm, *T* = 298 K. (B) TOF of photocatalytic oxygen evolution at different pHs vs irradiation time.

Finally, in liposomes with pH = 7, we can conclude that the catalyst dimer/trimer is a faster hole acceptor than the monomer, but this reaction is harmful for the total quantum efficiency of oxygen evolution because the oxidation of the catalyst dimer/trimer is one decomposition pathway.^51^ To investigate the effect of pH on the photocatalytic water oxidation in liposomes, this reaction was carried out at both pH = 4 and 7 under otherwise identical conditions (Figure 7). It is notable that the rates of dioxygen evolution at pH = 4 (TOF_max_ = 3.0 ± 0.6 min^–1^), where the dimer/trimer does not form, was almost twice as large as that at pH = 7 (TOF_max_ = 1.8 ± 0.2 min^–1^).

### Summary and Concluding Remarks

In summary, we observed two main differences when comparing the mechanism of homogeneous vs liposome photocatalytic water oxidation. First, the oxidative quenching between photosensitizer (PS) and electron acceptor (S_2_O_8_^2–^) is much less efficient in liposomes. The electrostatic interaction between positively charged PS molecules anchored at the surface of liposomes and the negatively charged electron acceptor leads to cooperative association and static quenching. For such oppositely charged “ion-pair” reactants, the initial photoproducts of oxidative quenching may be slower to escape from the solvent cage, resulting in undesirably fast charge recombination and lower quantum efficiency for the production of PS^+^. We expected to enhance the oxidative quenching efficiency by increasing the concentration of the electron acceptor, but increasing the bulk concentration of Na_2_S_2_O_8_ above 5 mM did not increase the quantum yield of charge separation, and we speculate that this may be because of the high local concentration of Na_2_S_2_O_8_ at the membrane-water interface, resulting in a saturated adsorption area. Another limitation to charge separation in liposome systems is unproductive self-quenching of PS* by neighboring ground-state PS molecules. Increasing the phospholipid/PS ratio to 40:1 reduced self-quenching, but this requires higher lipid concentrations, and with fewer PS per liposome and per catalyst the available light-harvesting antenna for each catalyst would be smaller.

A second main effect of supporting this photocatalytic water oxidation system on liposomes is a dramatic effect on the kinetics of hole transfer from the oxidized sensitizer to the catalyst. While in homogeneous environment PS^+^ undergoes bimolecular hole transfer to the catalyst at a reasonably high rate, in liposomes at pH = 4, the hole transfer rate was found to be several orders of magnitude slower, with the reaction occurring on the time scale of ca. 0.1–1 s. Such remarkably slow electron transfer does not prevent photocatalysis to occur because in peroxodisulfate-driven systems, the recombination between reduced acceptor and oxidized PS is prevented due to the irreversibility of the reduction of S_2_O_8_^2–^. In addition, although the hole transfer rate between PS^+^ and catalyst is slower in liposomes, it is probably still fast enough to keep up with the inefficient generation of PS^+^.

The apparent high-order rate dependence on [**3**] for hole transfer from PS^+^ can be assigned to the observed formation of dimer/trimer, also at pH = 4. We note that the concentration range of **3** employed here is 50–200 μM, which is lower than in a typical cyclic voltammetry experiment used to determine *E*^0^, and similar to that of the first photocatalytic report (216 μM),^31^ so this effect could have been important also in previous studies. Our findings suggest further studies of this interesting family of catalysts and its oxidation kinetics.

## Conclusions

Altogether, these mechanistic findings highlight the important modifications of the kinetics of photocatalytic molecular systems when going from homogeneous solutions to liposome membranes. They also provide interesting ideas for the optimization of liposome-supported photocatalytic water oxidation systems. First, charge separation, i.e., the generation of PS^+^, should be improved, for example, by controlling the surface charge of the membrane, the charge and amphiphilicity of the electron acceptor and PS, or by using secondary redox relays as primary quencher of PS* at the interface. Second, a better match needs to be found between the lipophilicity of the photosensitizer and that of the catalyst, which may favor their encounter within the membrane and thereby increase the rate of step 2. In the particular case of the ruthenium-based catalyst **2** used in this work, the dimer/trimer observed at pH = 7 seems to be much more prone to oxidation than the monomer, leading to fast catalyst decomposition that limits the overall stability of this system. However, this problem may be specific to this catalyst and may not occur with other catalysts based on, for example, first-row transition metals, which will be necessary if one wants molecular-hybrid material such as liposome-based systems to one day be used for solar fuel generation.

## Experimental Section

### Materials

All commercially available chemicals were used as received without further purification. Compounds **1b**, **3**, as well as amphiphilic C_17_ alkyl tail-functionalized photosensitizer **1a** and water oxidation catalysts **2** were synthesized according to published methods and are reported in the Supporting Information.^16,34,54−56^ A 50 mM phosphate buffer was prepared by dissolving a mixture of NaH_2_PO_4_·H_2_O and Na_2_HPO_4_·2H_2_O in deionized water to reach a final pH of 7.1 and 4.0 at room temperature.

### Preparation of the Liposomes

For the preparation of photocatalytically active liposomes, the amphiphilic photosensitizer **1a** in CH_3_OH and water oxidation catalyst **2** in CHCl_3_ were added in the desired mole ratio with the commercially available lipids 1,2-dimyristoyl-*sn*-glycero-3-phosphocholine (DMPC). A small amount of sodium 1,2-dimyristoyl-*sn*-glycero-3-phosphoethanolamine-*N*-[methoxy(polyethyleneglycol)-2000] (NaDSPE-PEG2K) was also mixed in 1:100 ratio relative to DMPC to avoid aggregation and stabilize the liposome dispersion in water. The organic solvents were carefully evaporated by N_2_ blowing until a film was obtained. The film was further dried under high vacuum for 1 h and subsequently hydrated by phosphate buffer (50 mM, pH = 7.1 or 4.0), followed by five successive cycles of freeze–thawing, using liquid N_2_ and water bath at 328 K (20 K above the transition temperature of DMPC). The freeze–thawed mixture was extruded 11 times with an Avanti Polar Lipids mini-extruder at 328 K and 200 nm cellulose membrane filters, yielding a clear solution and monodisperse liposomes.^57^ Dynamic light scattering confirmed a narrow size distribution of liposomes in all cases.

### Steady-State Absorption and Luminescence Quenching Measurements

UV–vis absorption spectra were recorded on a Cary 50 UV–visible spectrophotometer. Fluorescence titrations were performed by exciting the samples at 450 nm using a Fluorolog-3 instrument from Horiba Jobin–Yvon together with FluorEssence Software. Solutions of photosensitizer and sodium persulfate in homogeneous liposomes were prepared and stored in the dark to avoid photoreactions. All solutions were degassed with Ar before measurements. Spectral measurements showed that the emission intensity decreased by less than 5% during a second run.

### Nanosecond Transient Absorption Measurements

#### (Quanta-Ray)

For nanosecond transient absorption and kinetic emission measurements, optical excitation was performed using the third harmonic output of a frequency-doubled Q-switched Nd:YAG laser (Quanta-Ray Pro series, Spectra Physics) with 355 nm and passed through an OPO that was tuned to 460 nm, 10Hz, 8 mJ/pulse (in some cases 20, 30, and 50 mJ pulse^–1^). The pulse laser was coupled to an LP 920 detection system (Edinburgh Instruments) equipped with a pulsed XBO 450 W xenon Arc Lamp (Osram), which can provide the white light for probing. An iStar CCD camera (Andor Technology) and an LP920-K photomultiplier (PMT) detector connected to a Tektronix TDS 3052 500 MHz 5 GS/s oscilloscope were used for transient signal detection. Transient absorption data was acquired using LP 900 software and processed using Origin 2018 software.

#### (Brilliant B)

The samples were excited using the third harmonic output of Nd:YAG laser (Quantel, Brilliant B) with 355 nm and passed through an OPO that was tuned to 460 nm (15 mJ/pulse). The probe light was single wavelength and provided by an un-pulsed 150 W Xe lamp in a flash photolysis spectrometer (Applied Photophysics LKS.60). Two monochromators were used to minimize sample excitation by probe light, the first monochromator was set to the desired detection wavelength before reaching the sample, the second monochromator was placed after samples. The absorption difference of samples at a specified wavelength can be monitored by PMT Hamamatsu R928 detector and digitized using an Agilent Technologies Infiniium digital oscilloscope (600 MHZ). Transient absorption data was acquired within the Applied Photophysics LKS software package. All TA measurements were carried out at room temperature and a 1.0 cm path length quartz cell cuvette was used for the measurements, and before measurements, all solutions were degassed with Ar.

### Photo-Induced Oxygen Production

Photo-induced oxygen production from water was analyzed by a Clark oxygen electrode (Unisense OX-NP) controlled by x-5 UniAmp using Logger software. The irradiation source was an OSRAM Opto Semiconductors LD W5SM LED (λ_irr_ 450 nm, Δλ_1/2_ = 25 nm) with water cooling. All of the photochemical oxygen production measurements were carried out in a thermostated (298 K) photochemical reactor (total volume 25.0 mL) containing a 3.5 mL solution of liposome sample (10 μM **1a**, 5 μM **2**, and 100 μM DMPC) and Na_2_S_2_O_8_ (5 mM) in phosphate buffer (50 mM, pH 7.1 or 4). The system was degassed for 30 min with Ar, then data recording was started, first keeping the system in the dark for another 30 min before starting light irradiation.

## Supporting Information Available

Additional experimental details for the synthesis of the described metal complex; excitation energy-dependent emission lifetime of photosensitizer in liposomes; emission intensity and lifetime quenching of photosensitizer; transient absorption spectra of different systems in homogeneous environment; UV–vis spectra and additional flash photolysis data in homogeneous and liposomes system at pH 4, 7; photo-induced dioxygen production; and turnover number determination in liposomes at pH 4, 7 (PDF)
