# Supplementary material for: Mechanistic Insights into the Charge Transfer Dynamics of Photocatalytic Water Oxidation at the Lipid Bilayer–Water Interface
Source: J Am Chem Soc. 2022 Oct 17;144(42):19353–64. doi: 10.1021/jacs.2c06842 (PMC9619399; doi:10.1021/jacs.2c06842)
Supplement: Supplementary file 1 — ja2c06842_si_001.pdf [file ja2c06842_si_001.pdf]

Mechanistic Insights on Charge Transfer Dynamics of Photocatalytic Water Oxidation at the Lipid Bilayer-Water Interface

Hongwei Song,[a,] Agnese Amati,[b] Andrea Pannwitz,[b,c] Sylvestre Bonnet,[b] Leif Hammarström,*[a]

Institution Addresses:

[a] Department of Chemistry – Angstrom Laboratory, Uppsala University, Box 523, 751 20 Uppsala, Sweden

[b] Leiden Institute of Chemistry, Leiden University, Einsteinweg 55, 2333 CC, Leiden, The Netherlands

[c] Institute of Inorganic Chemistry I, Ulm University, Albert-Einstein-Allee 11, 89081 Ulm, Germany

Table of contents

Page 2-5 Synthesis of photosensitizer 1a and catalyst 2, 3

Page 6 Excitation energy dependent emission lifetimes of 1a in DMPC (1:10)

Page 6 Emission intensity and lifetime quenching of photosensitizer 1a, 1b

Page 7 Transient absorption spectra of photosensitizer 1b, 1b and $Na_2S_2O_8$, 1b, $Na_2S_2O_8$ and 3

Page 7 Flash photolysis results in homogenous environment in pH 7 and the Uv-vis spectrum of photocatalytic system before and after laser pulse in pH 4 and pH 7

Page 8 The Uv-vis spectrum of photocatalytic system in liposomes before and after laser pulse in pH 4 and pH 7

Page 8 Uv-vis spectrum of catalyst 2 in DMPC

Page 9 Flash photolysis results in liposomes in pH 7 with different concentration of catalyst 2

Page 9 Flash photolysis results in homogenous in pH 4 with different exposed time in air

Page 10-13 Photo-induced dioxygen production and turnover number determination

Synthesis of photosensitizer 1a and catalyst 2, 3

If not stated otherwise, all reagents and starting materials were purchased from commercial suppliers. $Ru(dmso)_4Cl_2$ [1] and the ligand N-dodecylisonicotinamide[2] were prepared according to published procedures.

A Bruker AV300/1 FT-NMR spectrometer was used to record ^{1}H-NMR, ^{13}C-NMR, as well as COSY, HSQC and HMBC 2D spectra. Mestre Nova was used for the evaluation of the spectra. The mass spectra were measured with a ThermoFischer Scientific MSQ Plus electrospray ionization mass spectrometer with a 17 – 2000 m/z detection range and a resolution of approximately 0.5 m/z. HR MS was measured via direct injection on a mass spectrometer (Thermo Finnagan LTQ Orbitrap) with electrospray ionization. Chromatographic silica columns were used for separating the components of the reaction mixtures, with a particle size of 40 – 63 µm and a surface area of 450 – 550 m^2/g. The pore volume of the particles was 0.75 – 0.85 cm^3/g. The silica powder was obtained from Screening Devices b.v.. Dry loading was carried out by adsorbing the mixture onto either celite or silica powder and solvent removal *in vacuo*, followed by deposition of the adsorbate on top of the column. The celite was obtained from Sigma-Aldrich. Elemental analysis was performed by Mikroanalytisches Laboratorium Kolbe in Oberhausen, Germany. The elemental content of the molecules was reported as the elements' mass fraction percentage.

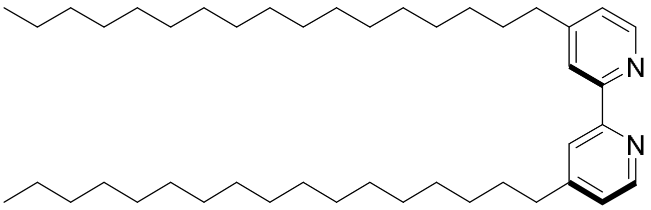

The synthesis of the ligand 4,4'-diheptadecyl-2,2'-bipyridine is adapted from a published procedure:[3] To diisopropylamine (4.05 mL, 2.92 g, 28.9 mmol, 2.66 eq.) in dry thf at 0 °C was added n-BuLi in hexane (2.5 M, 11.6 mL, 28.9 mmol, 2.66 eq.). This mixture was stirred at 0 °C for 1 h. At 0 °C 4,4'-dimethyl-2,2'-bipyridine (2.00 g, 10.9 mmol, 1.00 eq.) in dry thf was added via a syringe and stirring at 0 °C was continued for 3 h before adding hexadecyl bromide (8.82 g, 28.9 mmol, 2.66 eq.) in dry thf at this temperature. The mixture was stirred at room temperature for 3 days. The reaction mixture was poured onto ice-water (200 mL). The aqueous phase was extracted with diethyl ether (1x500 mL). The organic solvent was removed in vacuo and the solid was recrystallized from pentane (100 mL). Filtration and drying at air yielded 4,4'-diheptadecyl-2,2'-bipyridine as white solid (2.19 g, 3.46 mmol, 32 %).

^{1}H NMR (400 MHz, Chloroform-*d*) δ 8.55 (d, *J* = 5.0 Hz, 2H, C17bpy-6-H), 8.23 (d, *J* = 1.7 Hz, 2H, C17bpy-3-H), 7.13 (dd, *J* = 5.0, 1.7 Hz, 2H, C17bpy-5-H), 2.68 (t, 4H, α-CH_2), 1.68 (p, *J* = 7.4 Hz, 4H, β-CH_2), 1.25 (s, 56H, 28×CH_2), 0.87 (t, *J* = 6.7 Hz, 6H, CH_3). ^{13}C NMR (101 MHz, $CDCl_3$) δ 156.23 (C17bpy-2-C^q), 153.14 (C17bpy-4-C^q), 149.07 (C17bpy-6-H), 124.07 (C17bpy-5-H), 121.49 (C17bpy-3-H), 35.70 (α-CH_2), 32.07 (CH_2), 30.62 (CH_2), 29.84 (CH_2), 29.82 (CH_2), 29.80 (CH_2), 29.78 (CH_2), 29.68 (CH_2), 29.58 (CH_2), 29.51 (CH_2), 29.48 (CH_2), 22.84 (CH_2), 14.27 (CH_3). ESI-MS (MeOH) m/z calc. for $C_{44}H_{77}N_2^+$ $[M+H]^+$: 633.6, found: 633.6 (100 %). Elemental analysis calc. for $C_{44}H_{76}N_2$: C 83.47, H 12.10, N 4.42, found: C 83.43, H 12.07, N 4.39.

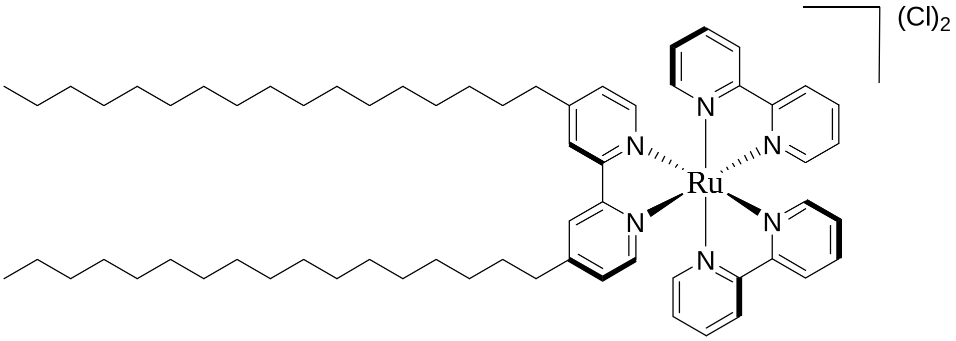

A mixture of [Ru(Cl)$_2$(bpy)$_2$] (754 mg, 1.56 mmol, 1.00 eq.) and 4,4'-diheptadecyl-2,2'-bipyridine (978 mg, 1.54 mmol, 0.99 eq.) in a 1/1/1 mixture of ethanol, water and chloroform (60 mL) were degassed via N_2 bubbling for 15 min and then heated at 110 °C for 2d. After cooling to room temperature, the solvent was removed in vacuo. The reaction mixture was subjected to column chromatography (SiO_2, acetone → 8/4/1 acetone:water:brine → 100/10/1 acetone/water/sat. $KNO_{3[aq]}$) to isolate the red/orange fraction. The organic solvent was removed in vacuo and the red compound was extracted with chloroform (3x). The combined organic layers were dried with $MgSO_4$ and the solvent was removed to dryness. The red solid was taken up in methanol and subjected to ion exchange column with Amberlite (50 g, pre-soaked with brine and washed 10x with water and 3x with methanol). The solvent was removed and the red solid was taken up in a mixture of chloroform and 1/1 water:brine. The phases were separated, and the aqueous phase was extracted with chloroform (2x). The combined organic layers were dried with $MgSO_4$ and the solvent was evaporated in vacuo. Trituration of the solid in acetone (100 mL) followed by removal of 50 mL of acetone at the rotavap, cooling to room temperature and filtration and washing with acetone (50 mL) yielded the desired compound as chloride salt (1·NaCl·3 H_2O) (1.14 g, 0.927 mmol, 60 %).

^{1}H NMR (400 MHz, MeOD) δ 8.67 (d, *J* = 8.2 Hz, 4H, bpy-3-CH, bpy-3'-CH), 8.59 (d, *J* = 1.9 Hz, 2H, C17bpy-3-CH, C17bpy-3'-CH), 8.15 – 8.03 (m, 4H, bpy-4-CH, bpy-4'-CH), 7.88 – 7.67 (m, 4H, bpy-6-CH, bpy-6'-CH), 7.61 (d, *J* = 5.8 Hz, 2H, C17bpy-6-CH, C17bpy-6'-CH), 7.46 (dtd, *J* = 7.2, 5.7, 1.3 Hz, 4H, bpy-5-CH, bpy-5'-CH), 7.31 (dd, *J* = 5.9, 1.8 Hz, 2H, C17bpy-5-CH, C17bpy-5'-CH), 2.82 (t, *J* = 7.9 Hz, 4H, α-CH_2), 1.71 (p, *J* = 7.3 Hz, 4H, β-CH_2), 1.44 – 1.11 (m, 56H, CH_2), 0.87 (t, *J* = 6.6 Hz, 6H, CH_3). ^{13}C NMR (101 MHz, MeOD) δ 158.63 (bpy-C^q), 158.60 (bpy-C^q), 158.19 (C17bpy-2-$C^{q,}$ C17bpy-2'-C^q), 156.56 (C17bpy-4-$C^{q,}$ C17bpy-4'-C^q), 152.65 (bpy-6-CH), 152.52 (bpy-6'-CH), 151.87 (C17bpy-6-CH, C17bpy-6'-CH), 139.01 (bpy-4-CH, bpy-4'-CH), 128.95(C17bpy-5-CH, C17bpy-5'-CH), 128.84 (bpy-5-CH, bpy-5'-CH), 125.67 (C17bpy-3-CH, C17bpy-3'-CH), 125.49 (bpy-3-CH, bpy-3'-CH), 36.26 (α-CH_2), 33.09 (CH_2), 31.35 (CH_2), 30.80 (CH_2), 30.77 (CH_2), 30.74 (CH_2), 30.63 (CH_2), 30.49 (CH_2), 30.44 (CH_2), 30.42 (CH_2), 23.75 (CH_2), 14.47 (CH_3). ESI-MS (MeOH) m/z (%): calculated for $[C_{64}H_{92}N_6Ru]^{2+}$: 523.3, found: 523.0. Elemental analysis calcd. for $C_{64}H_{92}Cl_2N_6Ru{\cdot}NaCl{\cdot}3H_2O$: C, 62.50; H, 8.03; N, 6.83, found: C, 62.63; H, 8.03, N 6.70.

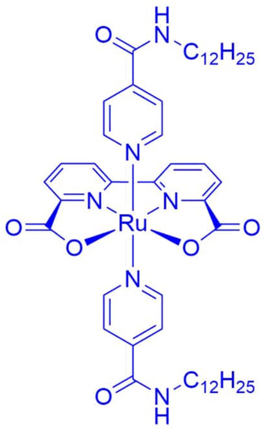

2

The synthesis of catalyst 2 was adapted form ref.:[2]

The degassed mixture of [Ru(Cl)$_2$(dmso)$_4$] (200 mg, 413 µmol, 1.00 eq.), [2,2'-bipyridine]-6,6'-dicarboxylic acid (101 mg, 413 µmol, 1.00 eq.) and triethyl amine (320 mL, 2.31 mmol, 5.60 eq.) in methanol (12 mL) was heated at reflux for 2 h. To the dark mixture was added a degassed solution of N-dodecylisonicotinamide (1.00 g, 3.44 mmol, 8.34 eq.) in methanol (12 mL) and the mixture was heated at reflux overnight. The solvent was removed. Degassed water (20 mL) was added to the solid, then degassed DCM (35 mL), and extraction was performed with degassed DCM (3x20 mL). The combined organic phases were dried with $MgSO_4$, and filtered off quickly. The solvent was removed and two column chromatographies (SiO_2, 200:8 → 200:15 → 200:30 DCM/MeOH) under N_2-atmosphere were performed to isolate the brown fraction that contained 2 · 1 CH_3OH (260 mg, 281 µmol, 68 %).

^{1}H NMR (300 MHz, Methanol-*d*$_4$, in presence of ascorbic acid) δ 8.66 (dd, *J* = 8.0, 1.2 Hz, 2H), 8.11 – 7.86 (m, 8H), 7.61 – 7.44 (m, 4H), 3.28 (d, *J* = 7.2 Hz, 4H), 1.61 – 1.48 (m, 4H), 1.38 – 1.17 (m, 36H), 0.96 – 0.81 (m, 6H). ESI-MS (MeOH) m/z (%): $[M+OH]^+$ calculated for $[C_{48}H_{67}N_6O_7Ru]^+$: 941.41, found: 941.4 (100 %), $[M]^+$ calculated for $[C_{48}H_{66}N_6O_6Ru]^+$: 924.41, found: 924.4 (45 %), $[M+CH_3O]^+$ calculated for $[C_{49}H_{69}N_6O_7Ru]^+$: 955.43, found: 955.2 (30 %). Elemental analysis calcd. for $C_{48}H_{64}N_6O_6Ru$ · 1 CH_3OH: C 61.55, H 7.38, N 8.79; found: C 61.78, H 7.21, N 8.76.

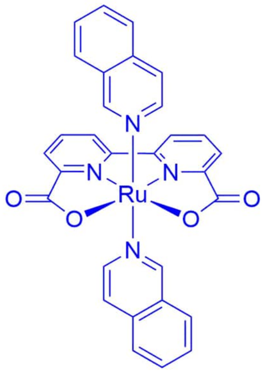

3

The synthesis of catalyst 3 was adapted form ref.:[4]

A mixture of 2,2'-bipyidine-6,6'-dicarboxylic acid (H_2bda) (25.2 mg, 103 µmol, 1.00 eq.), Ru(dmso)$_4$Cl$_2$ (50.00 mg, 103 µmol, 1.00 eq.) and triethylamine (0.2 ml) in methanol (15 ml) was degassed with N_2 and refluxed for 2 hours. An excess of isoquinoline (129 mg, 1.03 mmol, 10 eq.) was added and the reflux was continued overnight. The solvent was removed and the residual solid was purified by column chromatography on silica gel using degassed solvent mixture of DCM-methanol (1:0 to 10:1, v:v) as eluents, 3 was obtained as a brown solid (28 mg, 46 µmol, 45 %).

^{1}H NMR (400 MHz, methanol-d4/Chloroform-*d, 1:1* + *ascorbic acid*) δ 8.62 (d, *J* = 0.8 Hz, 2H), 8.52 (dd, *J* = 8.1, 1.1 Hz, 2H), 8.04 (dd, *J* = 7.7, 1.0 Hz, 2H), 7.86 (t, *J* = 7.9 Hz, 2H), 7.81 – 7.73 (m, 5H), 7.70 (ddd, *J* = 8.2, 6.8, 1.2 Hz, 2H), 7.60 (ddd, *J* = 8.2, 6.8, 1.3 Hz, 2H), 7.52 – 7.43 (m, 4H).

Excitation energy dependent emission lifetimes of 1a in DMPC (1:10)

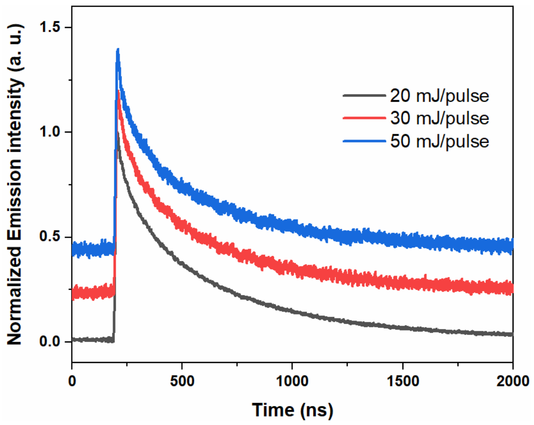

Figure S1. Normalized emission decay traces of 1a in liposomes (10:1 ratio) with different excitation energy. Conditions liposomes: 10 µM 1a, 100 µM DMPC, 1 µM NaDSPE-PEG2K in 50 mM phosphate buffer (pH=7). All solutions were purged with Ar before measurements at 20 °C. Excitation and detection wavelengths were fixed at 460 and 650 nm, respectively. For the red and blue traces, 0.2 and 0.4 offset are added for better visualization.

Emission intensity and lifetime quenching of photosensitizer 1a, 1b

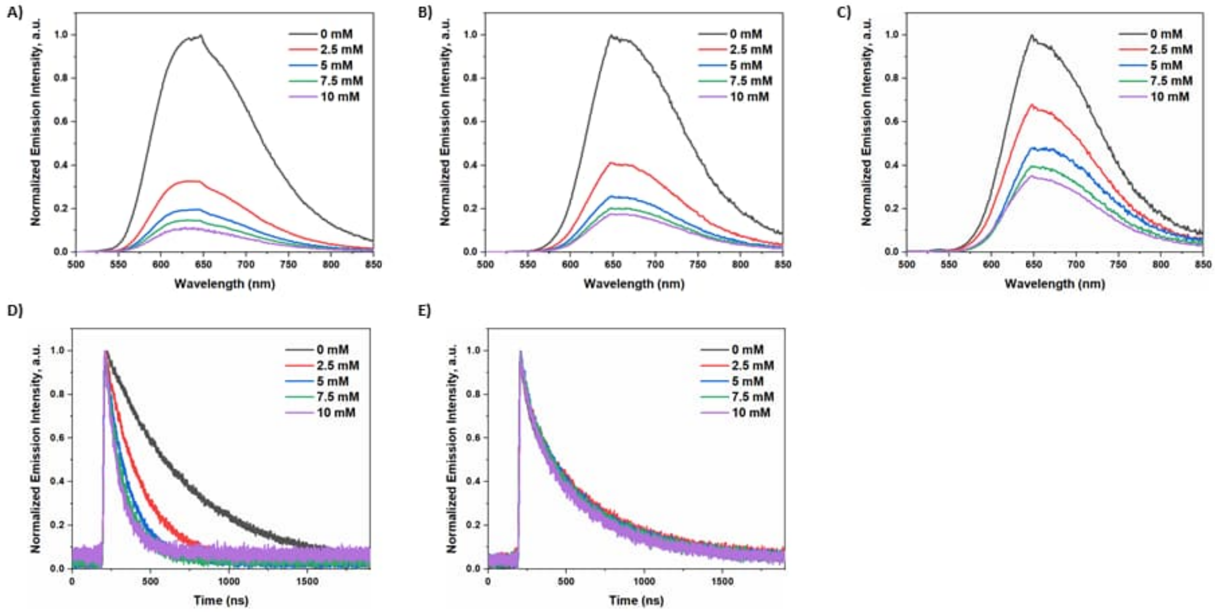

Figure S2. (A), (B), (C) Quenching of emission intensity of 1b and 1a by [$S_2O_8^{2-}$], determined by stationary fluorimetry (excitation at 450 nm). (D), (E) Emission decay traces of 1b and 1a, determined by pulsed experiments (excitation at 460 nm). Experimental conditions in homogenous environment: 10 µM 1b in 50 mM phosphate buffer (pH=7); Liposomes: 10 µM and 2 µM 1a, 100 µM DMPC, 1 µM NaDSPE-PEG2K in 50 mM phosphate buffer (pH=7). All solutions were purged with Ar before measurements at 20 °C.

Transient absorption spectra of photosensitizer 1b, 1b and $Na_2S_2O_8$, 1b, $Na_2S_2O_8$ and 3

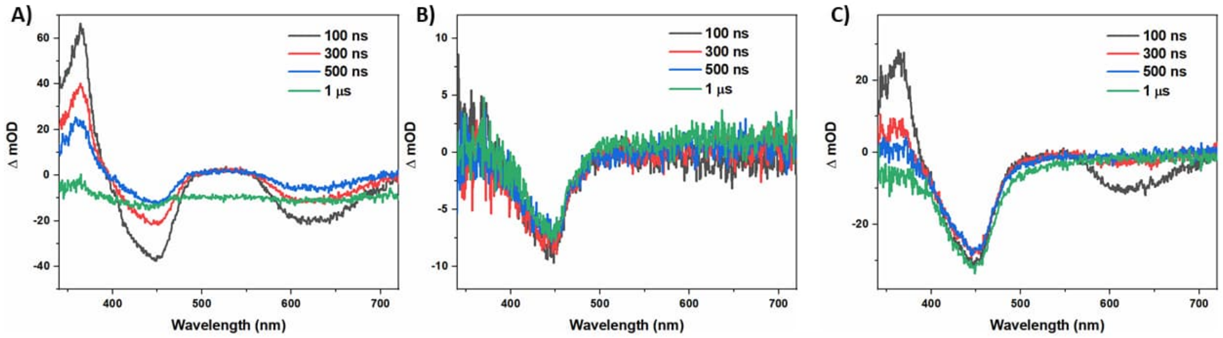

Figure S3. (A) UV-vis transient absorption spectra of 20 μM 1b obtained following 460 nm laser excitation (1 Hz, 10 mJ/pulse) in Ar-degassed buffer. (B) UV-vis transient absorption spectra of 20 μM 1b and 5 mM $Na_2S_2O_8$ obtained following 460 nm laser excitation (1 Hz, 10 mJ/pulse) in Ar-degassed buffer. (C) UV-vis transient absorption spectra of 20 μM 1b, 5 mM $Na_2S_2O_8$ and 100 μM 3 obtained following 460 nm laser excitation (1 Hz, 10 mJ/pulse) in Ar-degassed buffer.

Flash photolysis results in homogenous environment at pH = 7 and the Uv-vis spectrum of photocatalytic system before and after laser pulse at pH = 4 and pH = 7

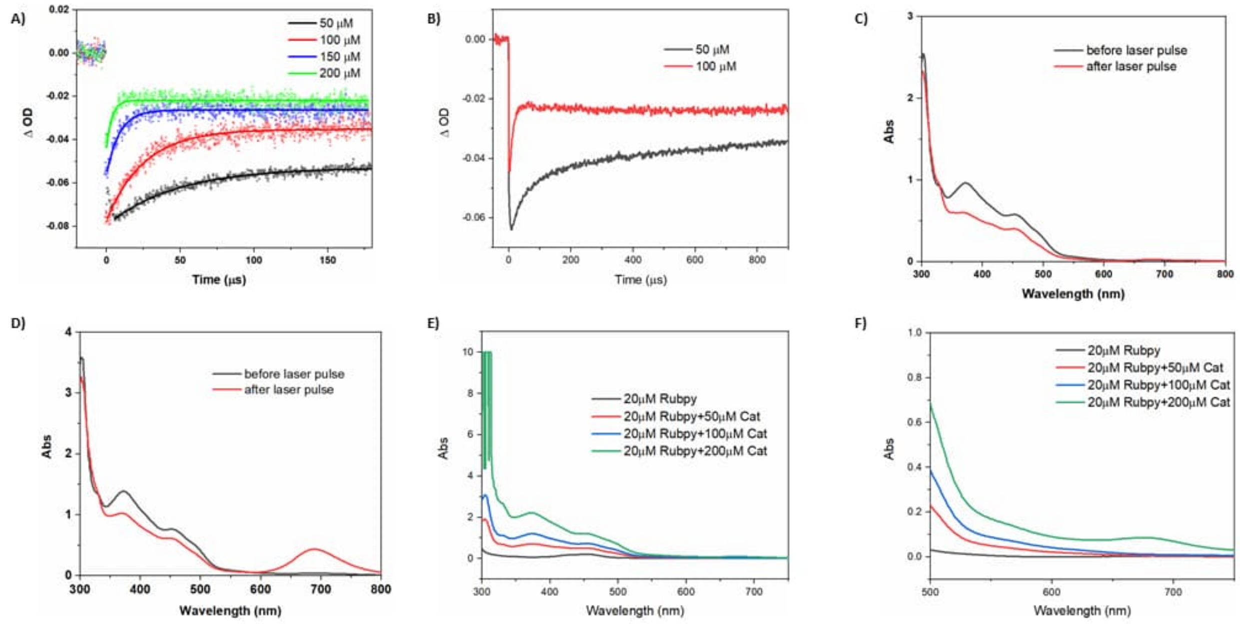

Figure S4. (A) Flash photolysis of aqueous solutions containing 20 μM 1b, 5 mM $Na_2S_2O_8$, 50 mM phosphate buffer (pH=7) and variable concentrations of catalyst at 450 nm. (B) The kinetic traces of 50 μM and 100 μM catalyst in longer time scale. (C), (D) Uv-vis spectrum of a solution containing 20 μM 1b, 5 mM $Na_2S_2O_8$, 100 μM 3, 50 mM phosphate buffer (pH=4 in B, pH=7 in D) before and after 8-ns 460-nm 10-mJ laser shot. (E), (F) Uv-vis spectrum of a 20 μM 1b and catalyst 3 with different concentrations in a 50 mM phosphate buffer (pH=4).

The Uv-vis spectrum of photocatalytic system in liposomes before and after laser pulse at pH = 4 and pH = 7

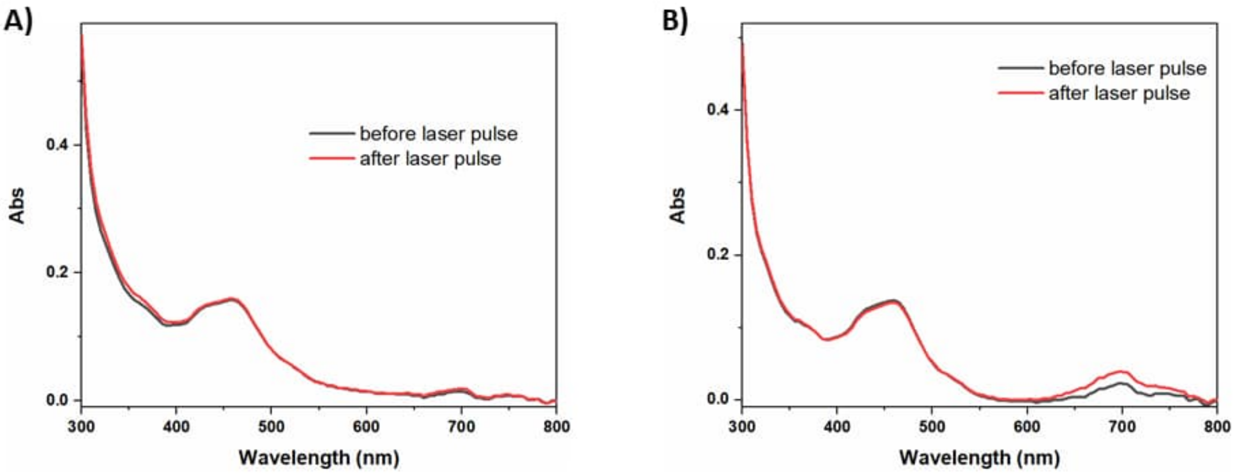

Figure S5. Uv-vis spectrum of a solution containing 10 µM 1a, 5 µM 2, 100 µM DMPC, 1 µM NaDSPE-PEG2K, 5 mM $Na_2S_2O_8$, 50 mM phosphate buffer pH=4 (A) and pH=7 (B) before and after laser 8-ns 460-nm 10-mJ laser shot.

Uv-vis spectrum of catalyst 2 in DMPC

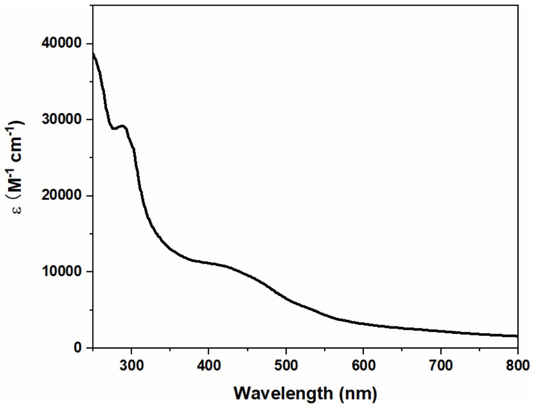

Figure S6. Uv-vis spectrum of a liposome solution made of 2, 100 µM DMPC, 1 µM NaDSPE-PEG2K, in a 50 mM phosphate buffer (pH=4), before laser shot.

Flash photolysis results in liposomes in pH 7 with different concentration of catalyst 2

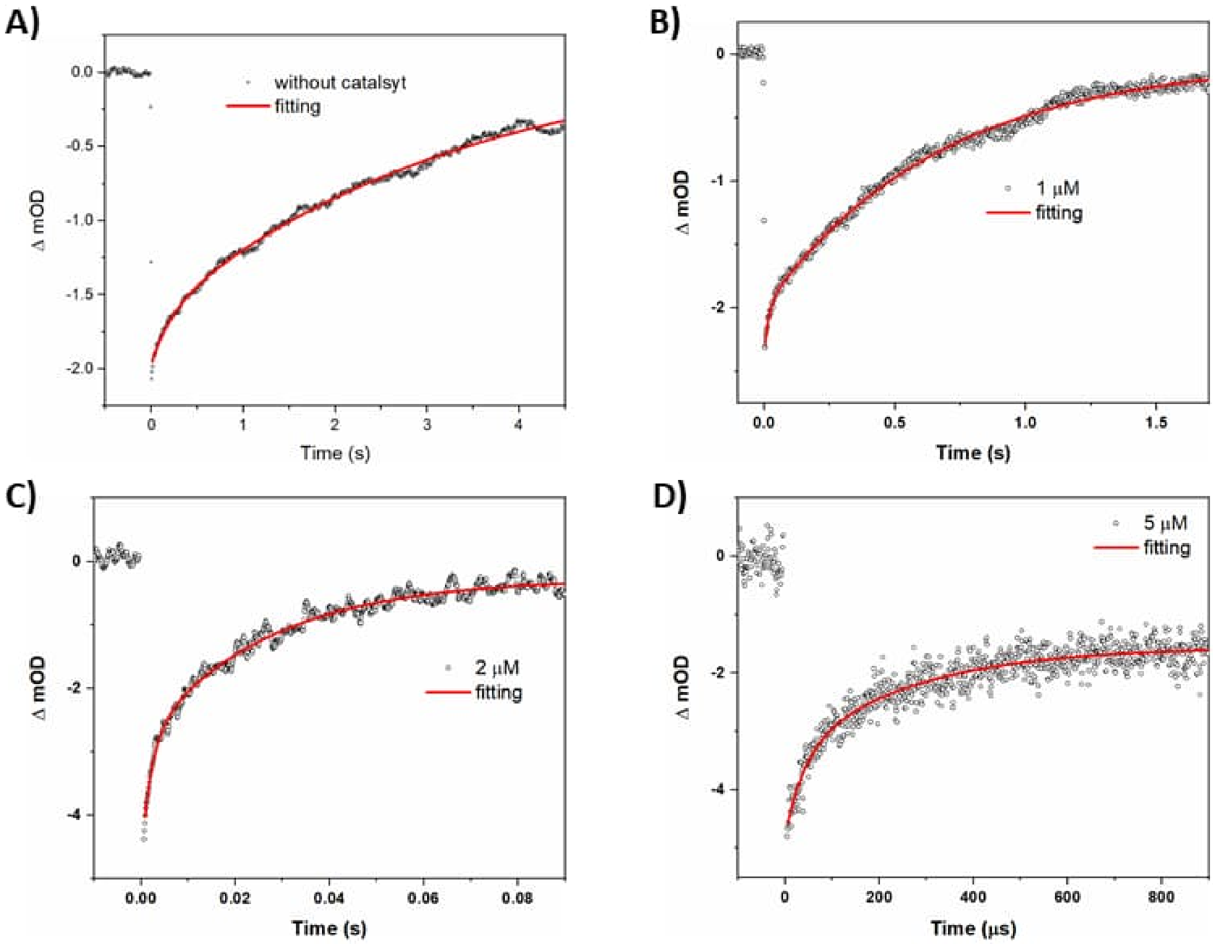

Figure S7. Flash photolysis at 450 nm of aqueous solutions containing 10 μM 1a, 0, 1, 2, 5 μM 2, 5 mM $Na_2S_2O_8$, 50 mM phosphate buffer pH=7. Excitation with 8-ns 460 nm pulse light.

Flash photolysis results in homogenous solution at pH 4 after different times of exposure to air

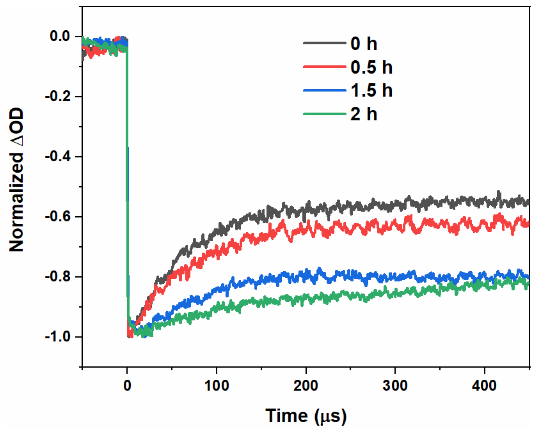

Figure S8. Flash photolysis kinetics at 450 nm of homogeneous, aqueous solutions containing 20 μM 1b, 100 μM 3, 5 mM $Na_2S_2O_8$, 50 mM phosphate buffer (pH=4) after different times of exposure to air. Excitation with 8-ns 460 nm pulse light.

Table S1. Time constants (τ) and relative amplitudes (a) obtained from fitting of the kinetic traces at 450 nm of photocatalytic system with 1a, 2 and 5 mM NaS_2O_8 at various concentrations of 2 in pH = 7 and pH = 4 phosphate buffer (traces in Figure S7 and Figure 6, respectively). The weighted average lifetime is calculated as $<\tau> = a_1\tau_1 + a_2\tau_2$.

	pH 7			pH 4		
[2] /μM	τ_1 (a_1) /s	τ_2 (a_2) /s	<τ> /s	τ_1 (a_1) /s	τ_2 (a_2) /s	<τ> /s
0	0.217(0.05)	3.3(0.95)	3.14			
1	0.016 (0.02)	0.67 (0.98)	0.67	0.03 (0.19)	0.42 (0.81)	0.35
2	0.002 (0.29)	0.026 (0.71)	0.02	0.01 (0.16)	0.18 (0.84)	0.15
5	$4 \cdot 10^{-5}$ (0.40)	$2.6 \cdot 10^{-4}$ (0.60)	$1.7 \cdot 10^{-4}$	0.01 (0.54)	0.10 (0.47)	0.06

Photo-induced dioxygen production

Photo-induced oxygen production from water was analyzed by a Clark oxygen electrode (Unisense OX-NP) controlled by x-5 UniAmp using Logger software. The irradiation source was an OSRAM Opto Semiconductors LD W5SM LED (λ_{irr} 450 nm, $\Delta\lambda_{1/2}$ = 25 nm) with water cooling.

Under constant stirring, the reactor was equipped with 1 rubber septum and 2 silicon septa in order to make an air-tight system (the set-up is shown in Figure S13). The Clark oxygen electrode was then inserted through the septum, to measure the dioxygen concentration in the head space (gas phase) of the photochemical reactor, and the whole system was deaerated by high-purity argon for at least 30 min. After removing the dioxygen, the Clark oxygen electrode was calibrated by a four-time injection of 100 μL (4.46 μmol at 1 atm) of high-purity O_2 into the closed system, thereby limiting the overpressure to <2%; the calibration was adapted with the pressure change using Logger software, affording direct reading of the volume of dioxygen (μL) produced in the gas phase of the reactor (Vgas = 21.5 mL).

All the photochemical oxygen production measurements were carried out in a thermostated (298 K) photochemical reactor (total volume 25.0 mL) containing a 3.5 mL solution of liposome sample (10 μM 1a, 5 μM 2, 100 μM DMPC, and 1 μM NaDSPE-PEG2K) and $Na_2S_2O_8$ (5 mM) in phosphate buffer (50 mM, pH 7 or 4). The system was degassed for 30 min with Ar, then data recording was started, first keeping the system in the dark for another 30 min before starting light irradiation.

Turnover number determination

The turnover number (TON) of oxygen evolution was determined by a Clark oxygen electrode (Unisense OX-NP) controlled by x-5 UniAmp using Logger software. The amount of oxygen formed during photoirradiation was used to calculate the TON. The TON were calculated from the oxygen production data by the following equation:

$$TON = \frac{n_{O_2}}{n_{cat}}$$

in which n_{O2} is the number of moles of dioxygen calculated from the volume of the dioxygen produced in the photocatalytic experiment as indicated by the calibrated Clark oxygen electrode in the gas phase

(µL), divided by 22.4 L/mol, and n_{cat} is the number of moles of catalyst used in the photocatalytic experiment.

The maximum turnover frequency TOF_{max} (in min^{-1}) of photocatalytic oxygen evolution was obtained using Origin 9.1 software by 1) nonlinear curve fitting of the time evolution of the TON (category: Growth/Sigmoidal, function: logistic Fit); 2) calculating the first derivative TOF = f(t) using mathematics, differentiate, and 3) identify the maximum value TOF_{max} of TOF = f(t) (Figure S11).

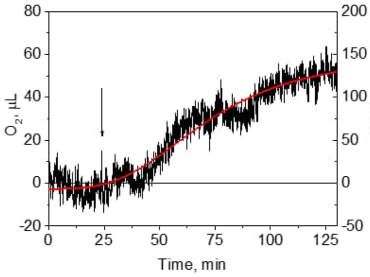

Model	Logistic		
Equation	y = A2 + (A1-A2)/(1 + (x/x0)^p)		
Reduced Chi-Sqr	28.94302		
Adj. R-Square	0.93552		
		Value	Standard Error
O2,uL	A1	-2.63336	0.33296
	A2	60.33704	1.01609
	x0	71.46322	0.92048
	p	3.10765	0.10081
	EC20	45.74543	0.51768
	EC50	71.46322	0.92048
	EC80	111.6394	2.78551

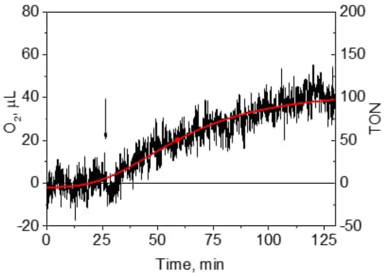

Model	Logistic		
Equation	y = A2 + (A1-A2)/(1 + (x/x0)^p)		
Reduced Chi-Sqr	27,30759		
Adj. R-Square	0,89622		
		Value	Standard Error
O2, uL	A1	-1,77605	0,35797
	A2	43,80551	0,54977
	x0	60,34899	0,77091
	p	2,8065	0,10074
	EC20	36,82533	0,67356
	EC50	60,34899	0,77091
	EC80	98,89934	2,46379

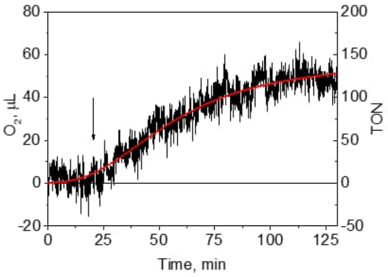

Model	Logistic		
Equation	y = A2 + (A1-A2)/(1 + (x/x0)^p)		
Reduced Chi-Sqr	28,75955		
Adj. R-Square	0,92046		
		Value	Standard Error
O2,uL	A1	0,40137	0,39648
	A2	57,70929	0,35119
	x0	56,50532	0,55971
	p	2,46281	0,06114
	EC20	32,18313	0,56429
	EC50	56,50532	0,55971
	EC80	99,20884	1,65814

Figure S9. Photocatalytic oxygen evolution at pH 7 vs. irradiation time (replicate experiments; the arrows show the minute at which irradiation was started), with non-linear curve fitting (and fitting parameter table). Conditions: 100 µM DMPC, 1 µM NaDSPE-PEG2K, 5 µM 2, 10 µM 1a, 5 mM $Na_2S_2O_8$ in 3.5 mL of phosphate buffer (50 mM, pH 7), $λ_{irr}$ 450 nm, T = 298 K.

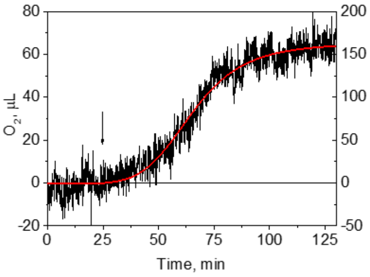

Model	Logistic		
Equation	y = A2 + (A1-A2)/(1 + (x/x0)^p)		
Reduced Chi-Sqr	27,47289		
Adj. R-Square	0,96118		
		Value	Standard Error
O2, uL	A1	-0,3294	0,25955
	A2	65,11657	0,43937
	x0	64,87518	0,3032
	p	5,54503	0,13323
	EC20	50,5245	0,32987
	EC50	64,87518	0,3032
	EC80	83,30192	0,71281

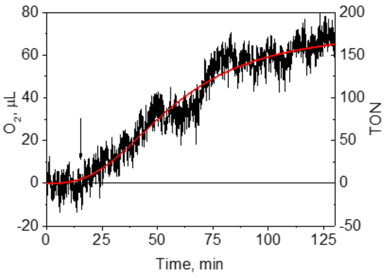

Model	Logistic		
Equation	y = A2 + (A1-A2)/(1 + (x/x0)^p)		
Reduced Chi-Sqr	40,79048		
Adj. R-Square	0,934		
		Value	Standard Error
O2, uL	A1	-0,03066	0,45689
	A2	71,92145	0,60118
	x0	56,6544	0,55502
	p	2,70566	0,07331
	EC20	33,94024	0,5149
	EC50	56,6544	0,55502
	EC80	94,56978	1,76217

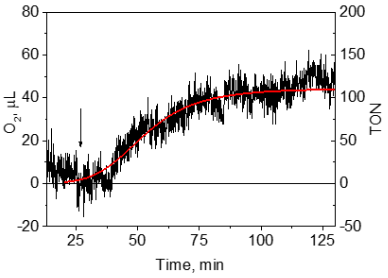

Model	Logistic		
Equation	y = A2 + (A1-A2)/(1 + (x/x0)^p)		
Reduced Chi-Sqr	15,62988		
Adj. R-Square	0,91053		
		Value	Standard Error
O2, uL	A1	0,3536	0,41489
	A2	44,50607	0,10231
	x0	52,41868	0,35148
	p	4,90173	0,1198
	EC20	39,50571	0,47164
	EC50	52,41868	0,35148
	EC80	69,55241	0,45583

Figure S10. Photocatalytic oxygen evolution at pH 4 vs. irradiation time (replicate experiments; the arrows show the minute at which irradiation was started), with non-linear curve fitting (and fitting parameter table). Conditions: 100 µM DMPC, 1 µM NaDSPE-PEG2K, 5 µM 2, 10 µM 1a, 5 mM $Na_2S_2O_8$ in 3.5 mL of phosphate buffer (50 mM, pH 4), λ_{irr} 450 nm, T = 298 K.

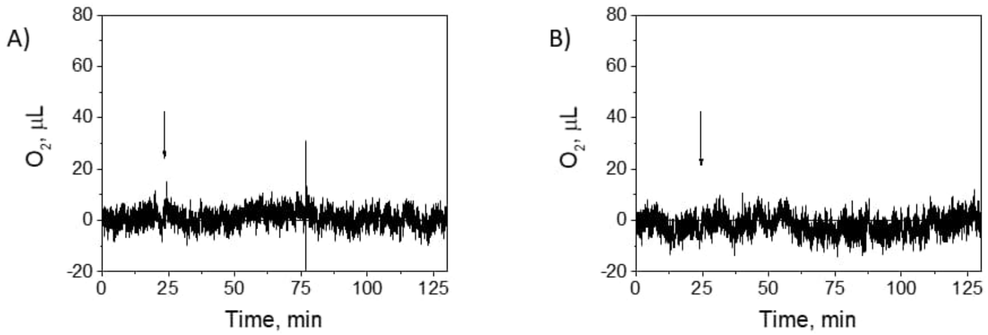

Figure S11. Control experiments without catalyst: (A) Photocatalytic oxygen evolution at pH 4 vs irradiation time. Conditions: 100 µM DMPC, 1 µM NaDSPE-PEG2K, 10 µM 1a, 5 mM $Na_2S_2O_8$ in 3.5 mL of phosphate buffer (50 mM, pH 4), λ_{irr} 450 nm, T = 298 K. (B) Photocatalytic oxygen evolution at pH 7 vs irradiation time. Conditions: 100 µM DMPC, 1 µM NaDSPE-PEG2K, 10 µM 1a, 5 mM $Na_2S_2O_8$ in 3.5 mL of phosphate buffer (50 mM, pH 7), λ_{irr} 450 nm, T = 298 K.

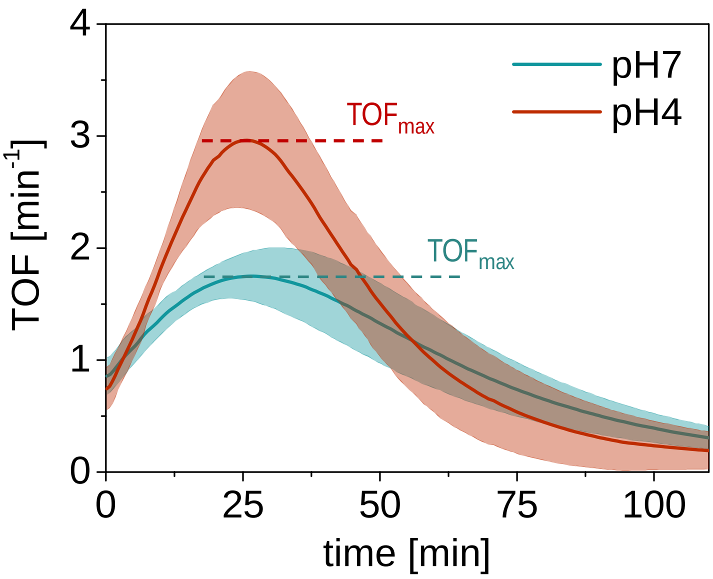

Figure S12. Calculation of maximum turnover frequency: TOF = f(t) is obtained as the first derivate of the curve fitting of the time evolution of the TON (red curves in Figure S9 and S10). Each curve is the mean of three replicate experiments, and the shaded areas show the standard deviations for each series.

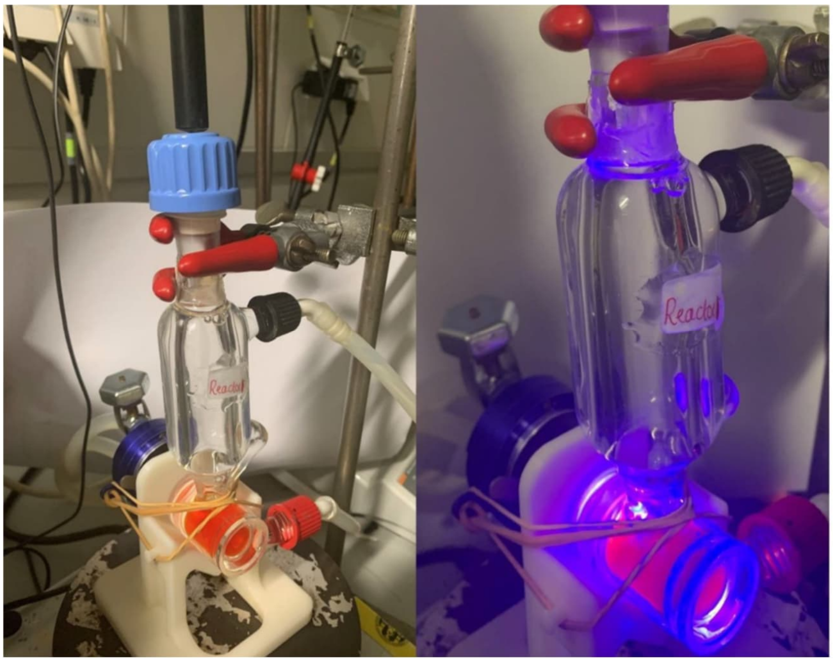

Figure S13. Photographs of the photocatalytic setup used for O_2 evolution experiments.

References

(1) Evans, I. P.; Spencer, A.; Wilkinson, G. Dichlorotetrakis(Dimethyl Sulphoxide)Ruthenium(II) and Its Use as a Source Material for Some New Ruthenium(II) Complexes. *J. Chem. Soc. Dalt. Trans.* 1973, No. 2, 204. https://doi.org/10.1039/dt9730000204.

(2) Limburg, B.; Wermink, J.; Van Nielen, S. S.; Kortlever, R.; Koper, M. T. M.; Bouwman, E.; Bonnet, S. Kinetics of Photocatalytic Water Oxidation at Liposomes: Membrane Anchoring Stabilizes the Photosensitizer. *ACS Catal.* 2016, *6* (9), 5968–5977. https://doi.org/10.1021/acscatal.6b00151.

(3) Neuthe, K.; Bittner, F.; Stiemke, F.; Ziem, B.; Du, J.; Zellner, M.; Wark, M.; Schubert, T.; Haag, R. Phosphonic Acid Anchored Ruthenium Complexes for ZnO-Based Dye-Sensitized Solar Cells. *Dye. Pigment.* 2014, *104*, 24–33. https://doi.org/10.1016/J.DYEPIG.2013.12.018.

(4) Duan, L.; Bozoglian, F.; Mandal, S.; Stewart, B.; Privalov, T.; Llobet, A.; Sun, L. A Molecular Ruthenium Catalyst with Water-Oxidation Activity Comparable to That of Photosystem II. *Nat. Chem.* 2012, *4* (5), 418–423. https://doi.org/10.1038/nchem.1301.
